# Different pH-sensitivity patterns of 30 sodium channel inhibitors suggest chemically different pools along the access pathway

**DOI:** 10.3389/fphar.2015.00210

**Published:** 2015-09-25

**Authors:** Alexandra Lazar, Nora Lenkey, Krisztina Pesti, Laszlo Fodor, Arpad Mike

**Affiliations:** ^1^Intensive Care Unit, University of Medicine and PharmacyTirgu Mures, Romania; ^2^Lendület Laboratory of Cellular Neurophysiology, Institute of Experimental Medicine, Hungarian Academy of SciencesBudapest, Hungary; ^3^Opto-Neuropharmacology Group, MTA-ELTE NAP BBudapest, Hungary; ^4^János Szentágothai Doctoral School of Neurosciences, Semmelweis UniversityBudapest, Hungary; ^5^Pharmacology and Drug Safety Research, Gedeon Richter Plc.Budapest, Hungary

**Keywords:** sodium channel blocker, pH, local anesthetic, antidepressant, automated patch-clamp

## Abstract

The major drug binding site of sodium channels is inaccessible from the extracellular side, drug molecules can only access it either from the membrane phase, or from the intracellular aqueous phase. For this reason, ligand-membrane interactions are as important determinants of inhibitor properties, as ligand-protein interactions. One-way to probe this is to modify the pH of the extracellular fluid, which alters the ratio of charged vs. uncharged forms of some compounds, thereby changing their interaction with the membrane. In this electrophysiology study we used three different pH values: 6.0, 7.3, and 8.6 to test the significance of the protonation-deprotonation equilibrium in drug access and affinity. We investigated drugs of several different indications: carbamazepine, lamotrigine, phenytoin, lidocaine, bupivacaine, mexiletine, flecainide, ranolazine, riluzole, memantine, ritanserin, tolperisone, silperisone, ambroxol, haloperidol, chlorpromazine, clozapine, fluoxetine, sertraline, paroxetine, amitriptyline, imipramine, desipramine, maprotiline, nisoxetine, mianserin, mirtazapine, venlafaxine, nefazodone, and trazodone. We recorded the pH-dependence of potency, reversibility, as well as onset/offset kinetics. As expected, we observed a strong correlation between the acidic dissociation constant (pKa) of drugs and the pH-dependence of their potency. Unexpectedly, however, the pH-dependence of reversibility or kinetics showed diverse patterns, not simple correlation. Our data are best explained by a model where drug molecules can be trapped in at least two chemically different environments: A hydrophilic trap (which may be the aqueous cavity within the inner vestibule), which favors polar and less lipophilic compounds, and a lipophilic trap (which may be the membrane phase itself, and/or lipophilic binding sites on the channel). Rescue from the hydrophilic and lipophilic traps can be promoted by alkalic and acidic extracellular pH, respectively.

## Introduction

The pH dependence of local anesthetic action has been observed almost a century ago, and the mechanism of pH dependence of sodium channel inhibitor properties has been studied for more than half a century (Ritchie and Greengard, 1966; Butterworth and Strichartz, 1990). From the clinical point of view, potency, distribution, and possible adverse effects of local anesthetics all largely depend on pH of the local tissue and/or the plasma (Lagan and McLure, 2004). From the theoretical point of view, pH-dependence of the inhibition may provide substantial insight into the mechanism of inhibition. Results of pH-dependence experiments led to the proposal of two alternative access pathways for local anesthetics to their binding site, a hydrophobic pathway through the membrane phase, and a hydrophilic pathway for the charged form of the molecules from the intracellular side of the membrane, through the activation gate of the channel. Both pathways require overcoming of a hydrophobic barrier (Narahashi et al., 1970; Hille, 1977a,b), which process is thought to require deprotonation. Because most local anesthetic molecules have an acidic dissociation constant (pKa) near physiological pH, the ratio of charged/protonated and uncharged/deprotonated forms is much dependent on pH. It has been observed that both onset and offset rates are accelerated by alkalic pH (Chernoff and Strichartz, 1990; Nettleton and Wang, 1990), and that the effect of pH is due to the deprotonation/protonation equilibrium of inhibitor drugs (Liu et al., 2003).

It has only recently been discovered, that beyond classic sodium channel inhibitor drugs, such as local anesthetics, type I antiarrhythmics and anticonvulsants, many drugs of other indications are also able to potently inhibit sodium channels (Huang et al., 2006), in fact sodium channels are among the most promiscuous targets (Lounkine et al., 2012). This presents the question, whether the binding site, access routes, and mechanisms proposed for pH-dependence originally described for local anesthetics and antiarrhythmics are generally true for all sodium channel inhibitors.

In this study we aimed to investigate a number of sodium channel inhibitors, which are of different therapeutical indications, and which have different chemical properties. We intended to investigate the following questions:

- Which chemical properties determine the pH-dependence of inhibition? Are there other important factors beyond lipophilicity (Bokesch et al., 1986; Courtney and Strichartz, 1987; Ehring et al., 1988; Brown et al., 1997, 1999; Wang et al., 2009; Desaphy et al., 2012), pKa (Courtney and Strichartz, 1987; Gerner et al., 2003; Liu et al., 2003), and size (Courtney, 1988, 1990)?
- Which specific processes might contribute to pH-dependence? Is deprotonation indeed the most important, and rate-limiting step?
- Which specific properties of inhibition are dependent on pH? We measured apparent affinity, reversibility, and onset/offset kinetics. Are these all equally affected by pH?

These questions have been previously studied for several different compounds, but a comparative study of a considerable number of diverse compounds has not yet been performed. In this study we compared the pH-dependence of inhibition by 30 different drugs, using the exact same experimental protocol, which, together with the analysis of their predicted chemical properties using a cheminformatics software, allowed the identification of some key chemical properties which determine pH-dependence of inhibition.

## Methods

Cell culture, electrophysiology and cheminformatics were carried out as in our previous study (Lenkey et al., 2010), where some aspects of the methodology are given in more details.

### Cell culture

HEK-293 cells stably expressing rNav1.2 sodium channels were obtained from NeuroSearch (Ballerup, Denmark). The cells were grown in Dulbecco's modified Eagle medium supplemented with 10% FBS. Prior to use, the cells were trypsinized and subsequently kept in suspension in the QPatch cell storage facility in CHO-S- SFM-II medium.

### Solutions

Composition of the extracellular solution was: (in mM): 140 NaCl, 3 KCl, 1 CaCl_2_ 1 MgCl_2_, 0.1 CdCl_2_, 20 TEA-Cl, 5 HEPES, adjusted to pH 7.3, Osmolality: 320 mOsm. The intracellular solution consisted of the following (in mM): 135 CsF, 10 NaCl, 1 EGTA, 10 HEPES, adjusted to pH 7.3 with CsOH (~5 mM), Osmolality: 320 mOsm. Drugs were synthesized in Gedeon Richter Plc. (paroxetine, lamotrigine, tolperisone, and silperisone), obtained from Tocris (mirtazapine, venlafaxine, nefazodone, flecainide ritanserin, and ambroxol), or Sigma (the rest of the compounds). Stock solutions were prepared at 10–100 mM in water, ethanol or DMSO, for the list of stock solutions see (Lenkey et al., 2010). The full list of drugs, with their three-letter codes, concentration, as well as their main therapeutic indication and main mechanism of action are shown in Table 1. In the case of two compounds, lidocaine and memantine we tested different concentrations, in order to verify our presumption that patterns of inhibition are due to the specific chemical properties of individual compounds, and are largely independent of concentration. The choice of concentrations used in this study may seem unusually high. The reason for this is, that in this study we did not aim to investigate close-to physiological inhibition mechanisms, but focused on studying the properties of access and egress pathways of the drugs instead. It is known that the affinity of sodium channel inhibitors is conformational state-dependent, and therefore it is also membrane potential-, use-, and frequency-dependent (Nardi et al., 2012). In a meta-analysis of 73 electrophysiology papers containing 246 individual measurements of 139 distinct compounds we found that the affinity to depolarized (open or inactivated) conformations was on an average 31-fold higher than to resting conformation (Lenkey et al., 2011). Because the 5 Hz protocol does not contain prolonged- or high frequency depolarizations, it gives IC_50_ values closer to resting affinity values. We chose concentrations (based on pilot studies) so that, if possible, the inhibition would be between 25 and 75% for all three pH values. We managed to achieve this in the case of 14 of the 30 compounds; for 13 compounds the inhibition was within the 25–75% interval for two out of the three pH values. From the remaining three compounds, in the case of silperisone, the choice of concentration was ideal, but the pH-dependence was too strong: 100 μM caused 23, 42, and 79% inhibition at pH 6.0, 7.3, and 8.6, respectively. The inhibition by venlafaxine was unexpectedly weak using this protocol, therefore even 100 μM turned out to cause less than 25% inhibition at pH values 6.0 and 7.3. Finally, in the case of flecainide, the unusually high concentration (300 μM) was necessary because of its unique mode of action: this drug can access open channels only (Anno and Hondeghem, 1990; Liu et al., 2002, 2003), therefore in this protocol it has a slow onset kinetics, especially at acidic pH.

**Table 1:**
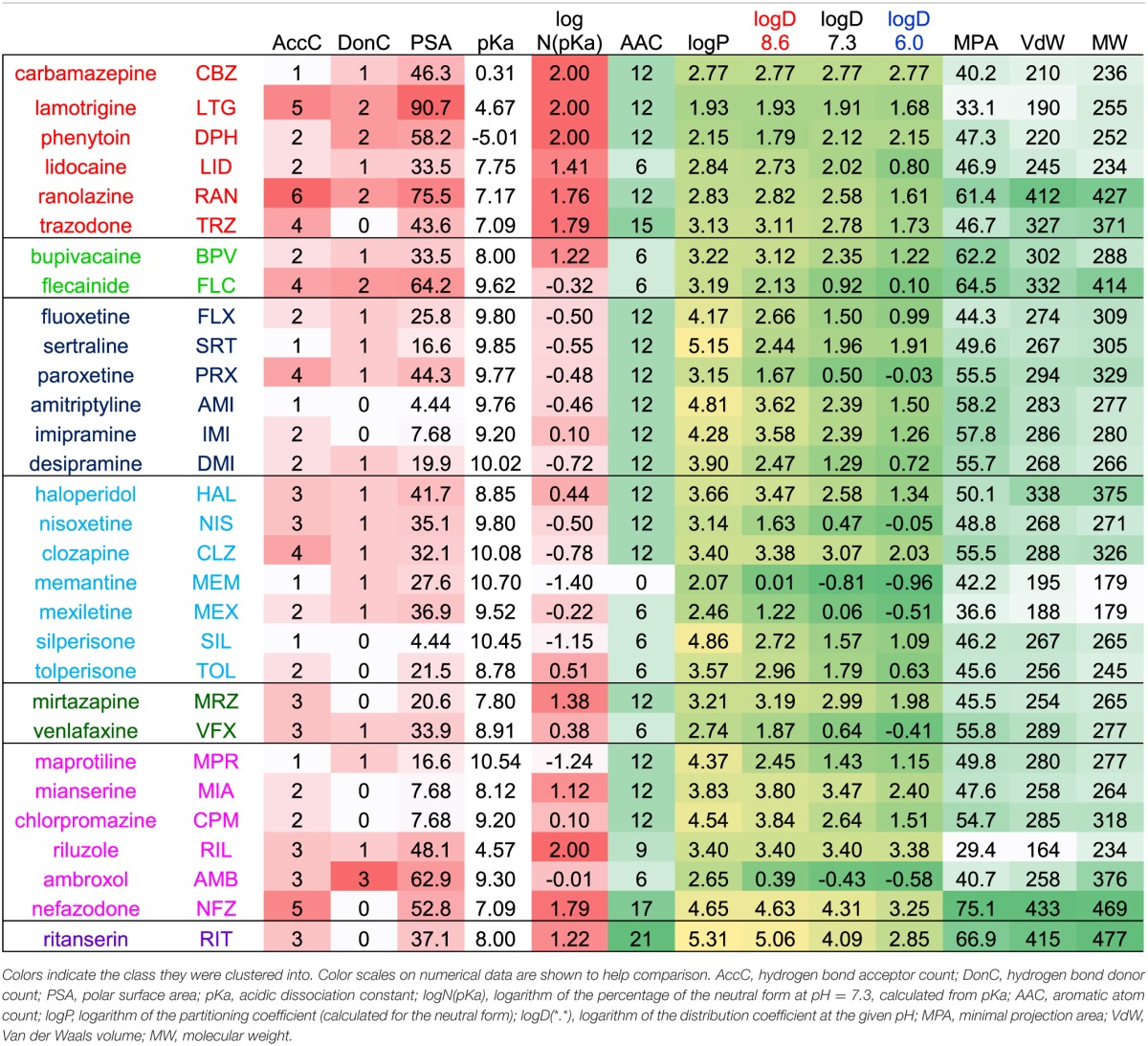
**Most important chemical properties calculated for the 30 compounds**.

Colors indicate the class they were clustered into. Color scales on numerical data are shown to help comparison. AccC, hydrogen bond acceptor count; DonC, hydrogen bond donor count; PSA, polar surface area; pKa, acidic dissociation constant; logN(pKa), logarithm of the percentage of the neutral form at pH = 7.3, calculated from pKa; AAC, aromatic atom count; logP, logarithm of the partitioning coefficient (calculated for the neutral form); logD(^*^.^*^), logarithm of the distribution coefficient at the given pH; MPA, minimal projection area; VdW, Van der Waals volume; MW, molecular weight.

### Electrophysiology

**Figure 1:**
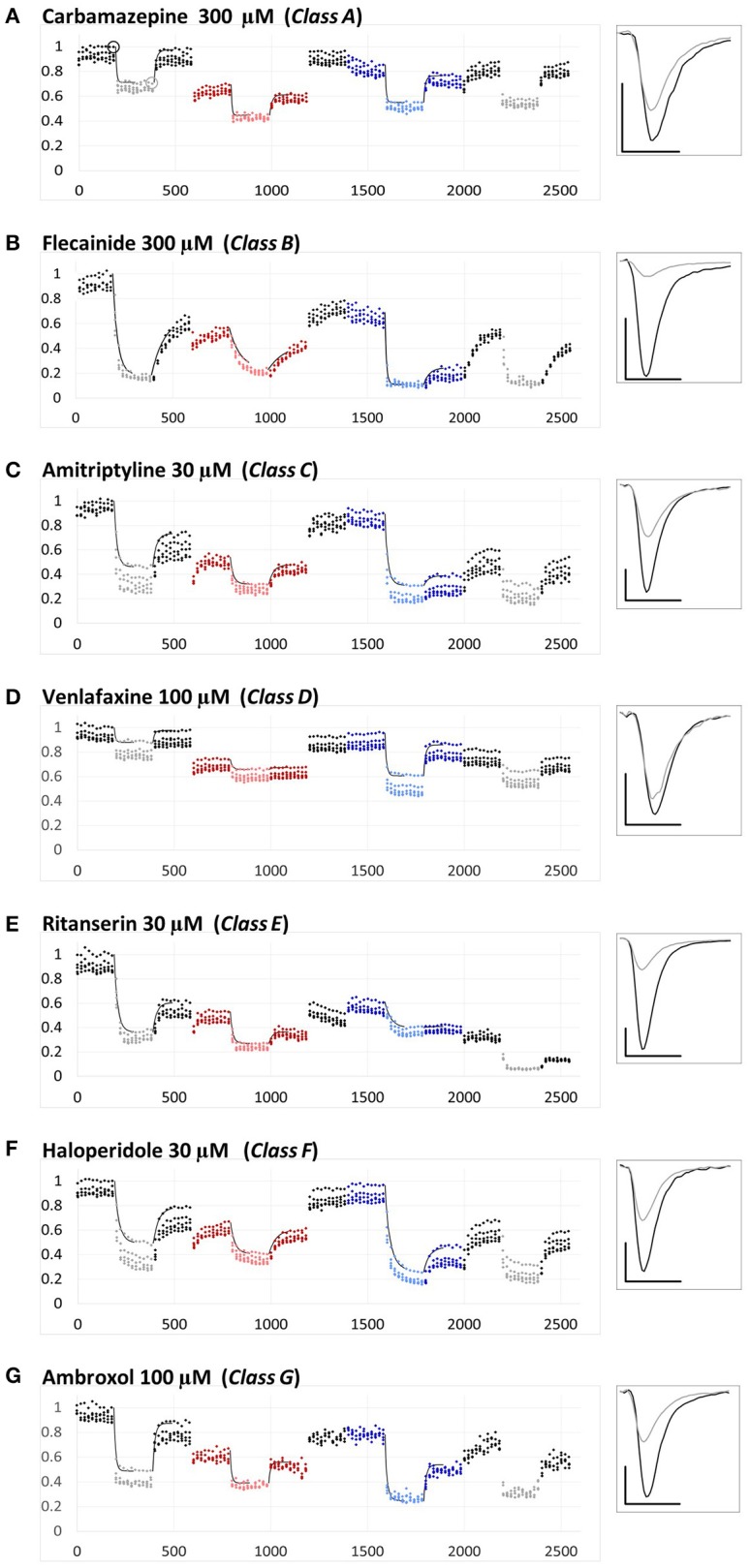
**Examples for the different types of pH-dependence patterns exhibited by the compounds**. Peak amplitudes are plotted against time during the whole experiment. Sodium currents were evoked by 5 Hz trains of 5 pulses, repeated in every 20 s. The pH of the perfusion medium was changed in the following order: neutral (black-gray-black)—acidic (red-pink-red)—neutral (black)—alkalic (dark-light-dark blue)—neutral (black-gray-black). Each major period consisted of 10 control trains, 10 trains during drug application (shown by light colors) and 10 trains during wash-out. Each plot shows the averaged normalized amplitudes of five individual experiments. All experiments were normalized to the amplitude of the current evoked by the first depolarization of the last train under control conditions. Thin black lines show the average of the five exponentials fit to individual curves, as described in Methods. Example traces from individual measurements are shown in the right panel. Black traces: Currents evoked by the first depolarization of the last control train [circled in **(A)]**. Gray traces: Currents evoked by the first depolarization of the last train during the first drug application period [circled in **(A)]**. Scale bars: 1 nA, 1 ms. **(A–G)** Examples for a member of each class from Class **(A–G)**.

All electrophysiological experiments were conducted on Qpatch-16 or QPatch HT instruments (Sophion, Ballerup, Denmark) using QPlate™ chips (Kutchinsky et al., 2003; Korsgaard et al., 2009; Danker and Möller, 2014). Data were sampled at a frequency of 25 kHz and filtered at 5 kHz. Junction potential was calculated to be −11 mV and was corrected for. Histograms for the distribution of electrical properties of cells (membrane resistance, series resistance, whole-cell capacitance, current amplitude), as well as activation and steady state inactivation curves are shown in the supplement of (Lenkey et al., 2010). Cells having membrane resistance < 500 MOhm, series resistance > 9 MOhm, or capacitance > 22 pF were excluded from analysis. The experimental protocol was also identical to the one used in our previous study: sodium channels were activated by 5 Hz trains of 5 depolarizations (−90 to −10 mV) repeated in every 20 s. In this study, however, the same control–drug-application–washout sequence was repeated four times: at neutral, acidic, alkalic, and again neutral pH. The peak amplitudes of sodium currents throughout the whole experiment are shown plotted against time in Figure 1 and Supplementary Figure 1.

### Data analysis and statistics

**Figure 2:**
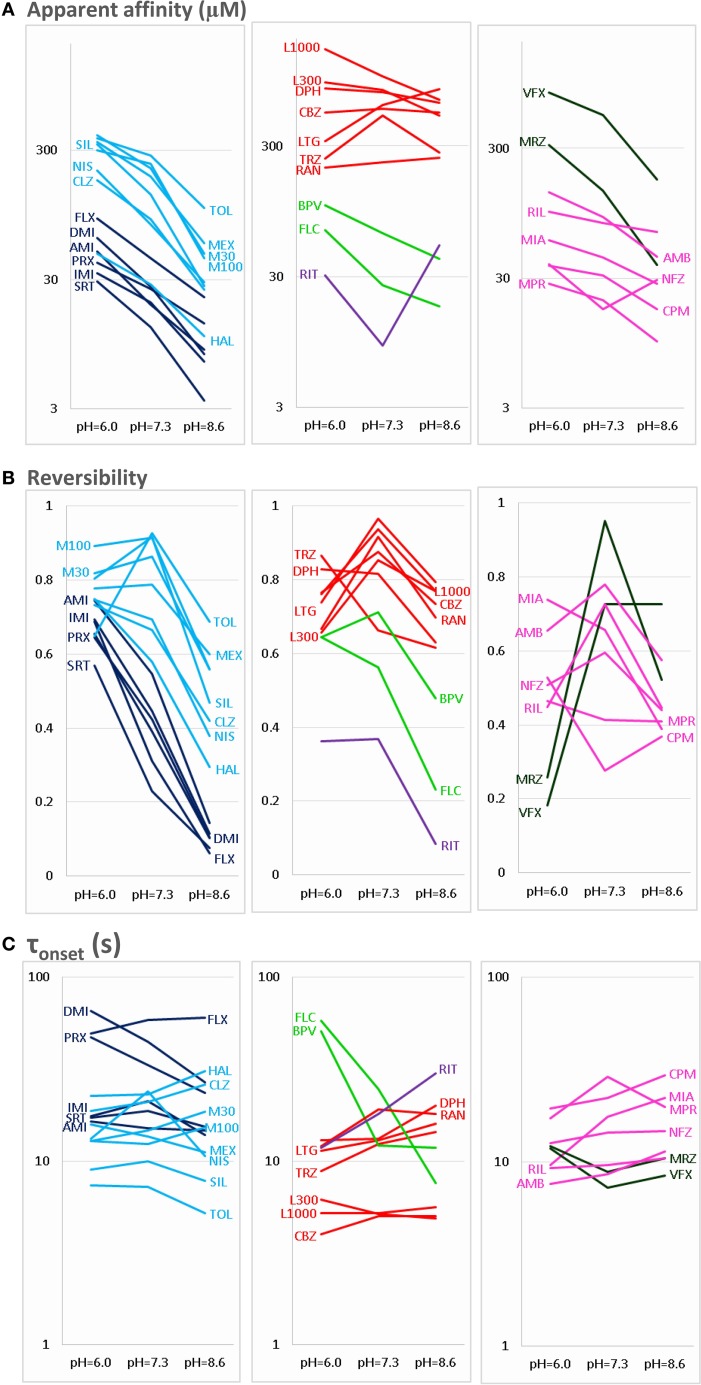
**pH-dependence of three properties of inhibiton**. The pH-dependence of **(A)** apparent affinity, **(B)** reversibility, and **(C)** onset time constant is illustrated for the 30 drugs. For the sake of clarity, the plots are divided into three parts: Left column shows Class C (dark blue) and Class F (light blue) compounds. Middle column shows Class A (red), Class B (light green), and Class E (purple) compounds. Right column shows Class D (dark green) and Class G (magenta) compounds. Identity of compounds is shown by the three-letter code, as shown in Table 1, except: M30 – memantine 30 μM, M100 – memantine 100 μM, L300 – lidocaine 300 μM, L1000 – lidocaine 1000 μM.

Initial analysis, including subtraction of leak and capacitive artifacts, as well as peak detection were done in the QPatch software. For further analysis and statistics Microsoft Excel (including Data Analysis Tools) and OriginPro 2015 (Originlab, Northampton, MA) were used. All experiments were normalized to the amplitude of the current evoked by the first depolarization of the last train under control conditions. Each plot shows the averaged normalized amplitudes of five individual experiments. For all four drug applications in an experiment, apparent affinity, onset and offset time constants as well as reversibility values were calculated. Apparent affinities (K_app_) were calculated from inhibition values as described in Lenkey et al. (2011), from the simplified Hill equation: one-to-one binding (i.e., n_H_ = 1) was supposed, so the Hill equation is reduced to Inh = cc/(cc + K_app_), from which K_app_ was calculated. (This method is equivalent to applying the Lineweaver-Burk plot method using a single concentration.) Reversibility values were calculated from control (c), inhibited (i), and wash-out (w) amplitudes as follows: Reversibility = (w-i)/(c-i). Reversibility values should not be regarded as experimental platform-independent properties of compounds. Recovery recorded in automated patch clamp systems, where solution flow is discontinuous (such as the QPatch instrument) is typically substantially lower than in manual patch clamp systems with continuous solution exchange [e.g., compare (Lenkey et al., 2010) with (Lenkey et al., 2006)], which is especially true for highly lipophilic “sticky” compounds (Danker and Möller, 2014). However, reversibility values are a valuable source of information regarding physicochemical differences between individual drugs, or between effects of the same drug under different extracellular pH conditions. Onset and offset time constants were determined by single exponential fitting to individual experiments. For neutral pH numerical data were given as the average of the data from the first and the last drug applications. Thin black lines on Figure 1 and Supplemental Figure 1 show the average of five fitted exponentials, (not the exponential fitted to the averaged normalized amplitudes). The numerical values of apparent affinity, onset and offset time constants and reversibility are shown in Table 2, and their relative position is illustrated in Figure 2. Affinity and time constant values were logarithmically transformed for statistics, therefore we show their geometric mean. For recovery data no transformation was done, and we show arithmetic mean values. Significance values were calculated using paired, two-tailed Student's *t*-test based on five pairs of apparent affinity (log transform), time constant (log transform), or reversibility values obtained from five individual experiments. Because of the large number of comparisons in this study, we accepted *p* < 0.01 as significant. Cluster analysis was done using Ward's minimum variance method, with Euclidean distance measure. Data were normalized by subtracting the mean (after logarithmic transformation in the case of apparent affinity and time constants), and dividing by the standard deviation. In order to prevent changing the sign of differences, difference values (pH = 6.0 vs. 7.3, 7.3, vs. 8.6 and 6.0 vs. 8.6) were normalized by only dividing by the standard deviation. Data for the cluster analysis included the three normalized apparent affinity values (at acidic, neutral and alkalic pH), the three normalized reversibility values, the three normalized onset time constants (offset time constants were not included, because at low recovery they were often ambiguous), and the difference values for all of these, altogether 18 variables. We have experimented with using different distance measures, replacing onset time constants with the average of onset and offset time constants, and assigning different weights (ranging between 1 and 2) to specific variables we considered more important, but these approaches did not radically change the overall classification, only the position of a few compounds (as we describe below). In the Results section, therefore, we will discuss the clusters obtained using the unweighted data with Euclidean distance measure.

**Table 2:**
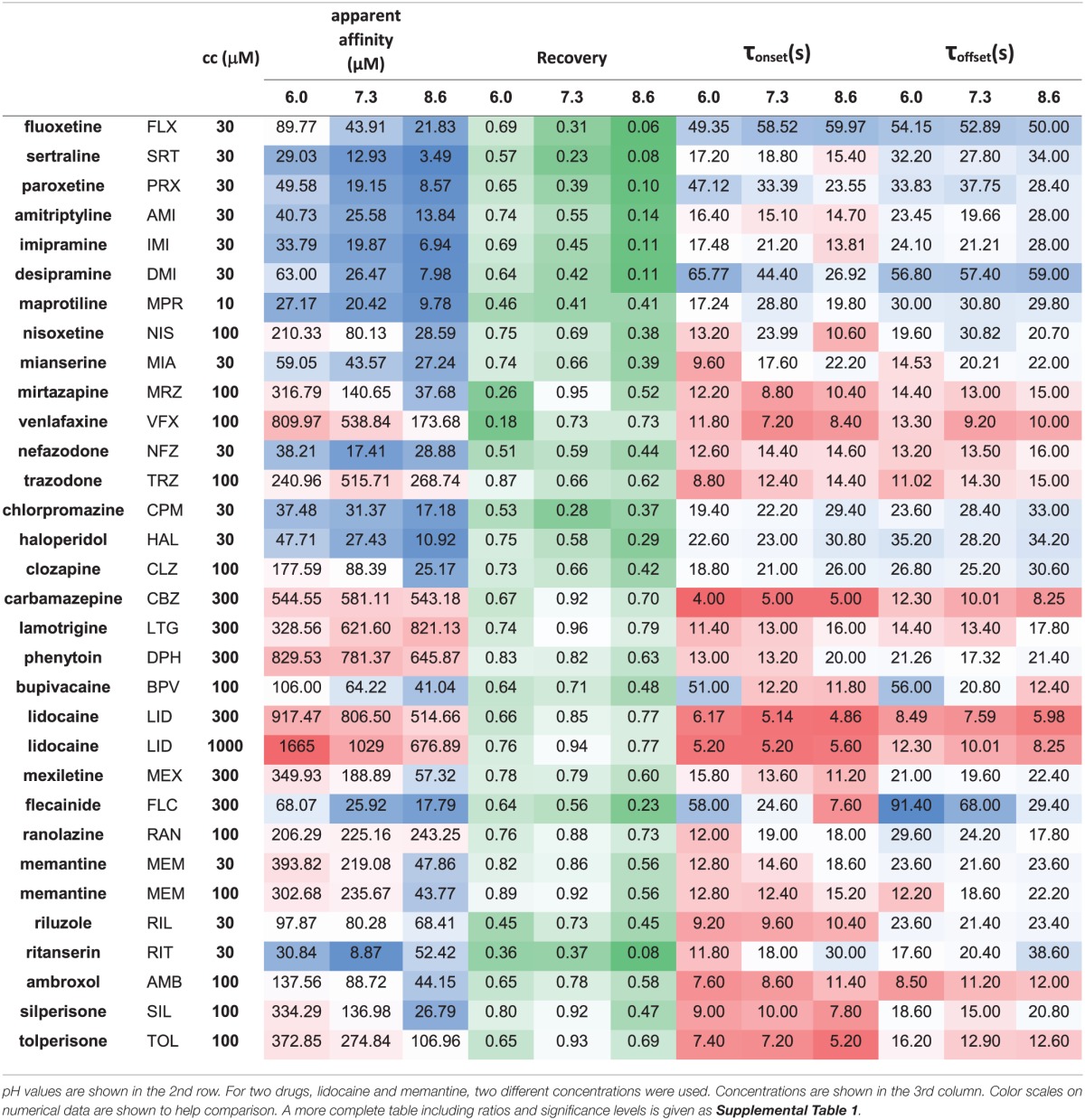
**Properties of inhibition measured for 30 drugs at 3 pH values**.

pH values are shown in the 2nd row. For two drugs, lidocaine and memantine, two different concentrations were used. Concentrations are shown in the 3rd column. Color scales on numerical data are shown to help comparison. A more complete table including ratios and significance levels is given as Supplemental Table 1.

### Cheminformatics

Chemical descriptors were generated using JChem for Excel 15.4 software from ChemAxon (Budapest, Hungary). Wherever the new version calculated descriptors differently from the earlier version (5.3.3) used in our previous studies (Lenkey et al., 2010, 2011), we used the values of the earlier version to ensure comparability. Based on the calculated descriptor values for the 30 drugs we created the correlation matrix for all descriptors in order to detect redundancies. Then together with all normalized properties of inhibition for the 30 drugs (which are: apparent affinity, reversibility, and onset/offset time constants for all three pH values, as well as the pairwise differences between pH values for all these properties; altogether 24 properties) we created the correlation matrix between chemical descriptors and properties of inhibition. Based on these correlation matrices we chose which of the descriptors are the most predictive and the least redundant. Lipophilicity is one of the most important properties, we expressed it using four different descriptors: the partition coefficient (logP) expresses the logarithm of octanol/water distribution of the compound's neutral form, while distribution coefficient (logD) considers all forms of the compound that are present at a specific pH. We calculated logD for three different pH values. The acidic dissociation constant had a skewed distribution, therefore in some of the plots we calculated from it the percentage of neutral molecules at pH = 7.3 using the rearranged Henderson-Hasselbalch equation: N(pKa) = 100^*^10^pH^/(10^pH^ + 10^pKa^). The logarithm of N(pKa) gave a fairly even distribution, and therefore it was used in several of the plots. Of the many descriptors that show the size of the molecule, we found the geometrical descriptors “minimal projection area” the most useful. This describes not the size but the “slimness” of the molecule, which is an important property for a binding site that can be accessed through a fenestration.

## Results

### General observations

Based on the literature of the external pH-dependence of sodium channel inhibitors, we expected to observe the following tendencies:

(a) For predominantly positively charged molecules the affinity was expected to be increased at alkalic pH (because deprotonation can help overcoming the hydrophobic barrier), while for predominantly neutral molecules no such effect was expected.
(b) The onset and offset of inhibition was expected to be accelerated at alkalic pH for predominantly positively charged compounds.
(c) Recovery from inhibition was expected to be incomplete at acidic pH, but accelerated by alkalic extracellular solution (Schwarz et al., 1977; Chernoff and Strichartz, 1990).

As regards point “(a),” in most but not all cases we have observed what was expected, while in the case of “(b)” and “(c),” we found important, unexpected differences from these presumptions for the majority of compounds. The pH-dependence of the extent of inhibition indeed seemed to be determined by the acidic dissociation constant (pKa) of individual compounds, i.e., by what percentage of the compound is in its charged vs. neutral form. However, as for the onset/offset rates, and the reversibility of inhibition, the relationship was more complex, and not solely determined by pKa. Most importantly, we found an obvious heterogeneity even among predominantly charged compounds (i.e., those with pKa values higher than 7.3), depending on their additional chemical properties. We have identified several distinct types of pH-dependence, and attempted to determine which specific chemical properties cause individual compounds to belong to a particular type. From the correlations between chemical properties and types of pH-dependence, we gained some insight into the details of the access pathway for inhibitor molecules to the binding site.

Using qualitative categories (like increase, decrease, or no change) we observed six distinct types of pH-dependence. Cluster analysis confirmed our preliminary classification, but quite dependably classified compounds into seven, not six categories. We will first introduce these seven major types of pH-dependence. Next, we will discuss their respective chemical properties. Obviously, these seven categories are artificial, because most chemical properties change along a continuum. We introduce them because this helps to discuss differences in a coherent way, and to understand major effects of chemical properties on properties of inhibition. It is necessary, however, to investigate these correlations irrespectively of subjective classification. This analysis will be presented in the last section.

The most important calculated chemical properties for all 30 compounds are shown in Table 1. One typical example of each major type of pH-dependence is illustrated in Figure 1. In Supplemental Figure 1. we show plots of peak amplitudes for all compounds.

### Major types of pH-dependent inhibition

The three anticonvulsant compounds, carbamazepine, phenytoin, and lamotrigine behaved similarly, and it was obvious from the observation of the data that they formed a separate group, which we named “***Class A.***” Since this group contained predominantly neutral molecules, their potency was not expected to be enhanced by alkalic pH. Furthermore, their onset/offset rate was expected to be fast, because deprotonation should not limit drug access to- and egress from the binding site, and the inhibition was expected to be reversible. Indeed, the extent of inhibition in this group was essentially independent of the pH, the onset/offset kinetics was fast, and reversibility was practically full at all three pH values (Table 2, red lines in Figure 2). To succinctly characterize Class A, it contains “*pH-independent-affinity—fast-onset—reversible*” compounds. Further characteristics of this group were the relatively low affinity (high apparent affinity values). The effect of 300 μM carbamazepine, a representative of Class A is shown in Figure 1A. The apparent affinity of lamotrigine was in fact not totally pH-independent, but showed an “inverse” pH-dependence, being most potent at acidic pH. When considering all its properties, however, it obviously belonged to this class. We investigated which other compounds might belong here by performing cluster analysis, and found that three additional compounds, trazodone, ranolazine, and lidocaine were sorted into Class A. It was evident that acidic dissociation constant (pKa) was the single most important factor which determined whether or not a compound belonged to Class A. From the eight compounds with the lowest pKa values, six was clustered here, with the exception of riluzole and nefazodone. Both riluzole and nefazodone had higher affinity than the group's average, which probably was the main reason why cluster analysis placed them into other groups. It was surprising that lidocaine too was clustered with the anticonvulsants and not with other antiarrhythmics, even though its apparent affinity did have a moderate (1.78- and 2.46-fold for 300 and 1000 μM lidocaine, respectively), but significant (*p* < 0.01) pH-dependence. However, lidocaine has the lowest pKa (7.75; i.e., 26% is neutral at pH = 7.3) among the investigated antiarrhythmics, and it had the fastest kinetics, highest recovery and lowest affinity. All these properties make its inhibition pattern similar to those of the three anticonvulsants. In our previous study, where we recorded other properties of inhibition, lidocaine had also been found to be closer to these anticonvulsants than flecainide or bupivacaine [see Figure 2 of (Lenkey et al., 2010)]. However, we must emphasize that the protocol we used in this study investigates only one aspect of the inhibition mechanism, and therefore similar inhibition patterns in this test do not prove similar mode of action. Amplitude plots showing the effect of phenytoin, lamotrigine, trazodone, ranolazine, and lidocaine are shown in Supplemental Figure 1.

Only the two antiarrhythmic compounds flecainide (Supplemental Figure 1) and bupivacaine (Figure 1B) behaved exactly as expected from the literature: The pH dependence of inhibition was not only reflected in apparent affinities and in reversibility, but also in the kinetics of inhibition. A definite acceleration of both onset and offset was evident at alkalic pH. This group was therefore named “*pH dependent affinity—pH dependent kinetics—pH dependent reversibility*” group; ***Class B*** (light green lines in Figure 2). No other compound clustered together with these two antiarrhythmics. Although the pH-dependence of mexiletine was qualitatively similar, in terms of reversibility and kinetics it was weaker, with significance levels *p* = 0.06 and *p* = 0.03, respectively (Supplemental Table 1).

Tricyclic (amitriptyline—Figure 1C, imipramine and desipramine), and selective serotonin reuptake inhibitor (fluoxetine, sertraline, and paroxetine) antidepressants formed a separate “*pH dependent affinity—slow kinetics—strongly pH dependent recovery*” class (***Class C***). Amplitude plots are shown in Supplemental Figure 1, the properties of this group, as compared to others are illustrated in Figure 2 (dark blue lines). The most obvious sign of belonging to this class was the extreme pH-dependence of recovery; while at pH = 6.0 it ranged between 0.57 and 0.74, at pH = 8.6 it was less than 0.14 for all six compounds. The onset time constant at pH = 7.3 was in the range of 15–59 s. In the case of desipramine and paroxetine, but not the rest of the compounds, a significant acceleration of onset was also observed at higher pH. (Quantitative properties are given in Table 2, and illustrated in Figure 2).

Two antidepressant compounds, venlafaxine (Figure 1D) and mirtazapine (Supplemental Figure 1; dark green lines in Figure 2) were exceptional because their reversibility was almost complete at pH = 7.3, but their effect was virtually irreversible at pH = 6.0. Decreased reversibility at pH = 6.0 was not at all uncommon among compounds of all classes, but only for these two compounds was the difference significant at *p* < 0.01 level. Their group, ***Class D***, can be characterized as “*pH dependent affinity—fast kinetics—low acidic recovery*” group.

The inhibition pattern shown by the anxiolytic ritanserin (Figure 1E, purple line in Figure 2) was totally different from the inhibition caused by all the rest of the drugs we have studied, with the possible exception of nefazodone. First, ritanserin was the only drug that was most potent at neutral pH (3.5-fold more potent than at pH = 6.0 (*p* < 0.01), and 5.9-fold more potent than at pH = 8.6 (*p* < 0.01); although nefazodone showed similar tendency, the differences were nonsignificant—see Supplemental Table 1). Second, in spite of being the most potent at neutral pH, it was also the most reversible at this pH (*p* < 0.001 between pH = 7.3 and 8.6); for nefazodone the differences were significant at *p* = 0.023, and 0.062 when pH = 7.3 was compared to pH = 6.0 and 8.6, respectively. Third, ritanserin showed the largest, and most significant deceleration of both the onset and the offset at alkalic pH (nefazodone showed only a slight, non-significant tendency). Cluster analysis, which is based on quantitative data in most trials did not classify the two drugs into the same group, therefore ritanserin remains the only member of ***Class E***, which is characterized as the group of “*most potent at neutral pH—decelerated by alkalic pH—low alkalic recovery*” compounds. Although nefazodone was in most trials clustered into Class G, (see below) we think it appropriate to call attention to the qualitative similarity of its inhibition pattern to that of ritanserin. Note that ritanserin was the most lipophilic of the 30 drugs (logD (7.3) = 4.45, logP = 5.31—in fact it was the only “non-drug-like” compound according to Lipinski's rule of five), and also the one with the highest molecular weight (MW = 478 g), furthermore it had the second highest minimal projection area (66.9 Å^2^), and the highest aromatic atom count (AAC = 21). Chemical properties of nefazodone were similar: it was the molecule with the highest minimum projection area (75.1 Å^2^) and distribution coefficient [logD (7.3) = 4.40], as well as the second highest molecular weight (MW = 470 g) and aromatic atom count (17). These large, strongly lipophilic molecules seemed to have behaved differently under these experimental conditions, and even might have had different binding site(s) than the rest of the drugs.

The two remaining classes contained compounds of moderate reversibility (0.29–0.68), and moderate pH-dependence of both apparent affinity and reversibility (light blue lines in Figure 2). ***Class F*** contained “*pH-dependent affinity—intermediate kinetics—alkalization dependent recovery*” compounds, these drugs had apparent affinity ratios (pH = 7.3 vs. 8.6) between 2.51 and 5.38, and their reversibility was also strongly sensitive to alkalization. The compounds sorted into this class were haloperidol nisoxetine, clozapine, mexiletine, memantine, silperisone, and tolperisone (Supplemental Figure 1).

***Class G*** compounds showed only weak pH-dependence of affinity, with pH = 7.3 vs. 8.6 ratios being between 0.6 and 2.09. This group of compounds could be characterized as “*moderately pH-dependent affinity—intermediate kinetics—moderately pH-dependent reversibility.*” It is a rather heterogeneous group, containing some compounds (e.g., riluzole, nefazodone) that have been shown to act by clearly different mechanisms of action using other experimental protocols (Lenkey et al., 2010, and unpublished data). Nevertheless, they are all similar in their moderate pH-dependence. Ambroxol (Figure 1G), maprotiline, mianserin, chlorpromazine, riluzole, and nefazodone (Supplemental Figure 1) were sorted into this class.

Results of cluster analysis, of course, are not to be considered indisputable; therefore we performed multiple analyses as described in the Methods section. Most analyses created the same clusters, but for three of the compounds the classification was uncertain. Haloperidol was sorted into Class C in some of the analyses, probably because of its high affinity and slow kinetics. Tolperisone had very fast kinetics, and it showed highest recovery at neutral pH, which properties caused it to be closer to Class A. However, considering the definite pH-dependence of its affinity, we are confident that it rather belongs to Class F. Finally, the weak pH-dependence of apparent affinity placed mianserin into Class G, but because of its strongly pH-dependent reversibility, it was occasionally sorted into Class F. The main properties of the classes are summarized in Table 3.

**Table 3:** **Qualitative properties of pH-dependence classes**.

	**K**_**D app**_		**Reversibility**		**τ**_**onset**_	
	**Acidic**	**Alkalic**	**Acidic**	**Alkalic**	**Acidic**	**Alkalic**
Class A	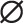	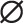	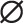	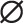	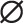	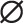
	LID ↑	LID ↓				
Class B	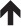	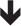	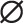	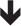	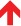	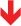
Class C	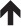	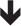	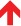	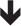	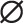	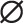
					DMI,PRX 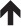	DMI,PRX 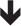
Class D	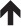	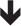	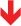	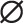	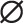	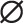
Class E	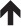	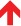	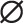	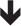	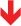	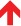
Class F	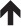	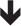	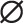	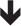	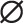	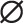
	MEM,MEX,TOL↑					
Class G	↑	↓	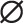	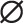	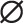	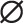
		NFZ 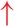		MIA↓		

Summary of the observed qualitative differences between different classes of pH-dependence. The effect of acidic (pH = 6.0) and alkalic (pH = 8.6) medium is shown on three properties of inhibition: apparent dissociation constant (KD app), reversibility and onset time constant. Bold arrows (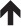

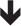
) indicate substantial modification, thin arrows (↑↓) indicate weak modification, 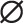 sign indicates no significant effect. Note that increased apparent dissociation constant (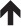) indicates decreased apparent affinity and vice versa. Similarly, increased onset time constant (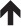) indicates decelerated onset and vice versa. Occurrence of exceptions is indicated at the bottom of the cells. Distinguishing features are in red fonts.

### Chemical properties of classes

We have detected different types of pH-dependent inhibition patterns. The question is, if we can explain why different classes behave differently, and if we can predict the pH-dependent behavior of specific compounds from chemical structure.

We address these questions in two consecutive steps. First we show that key chemical properties indeed seem to determine the type of pH-dependence. We illustrate the position of the classes introduced above in the chemical space. For this to perform, it is necessary to simplify the n-dimensional chemical space, and to find the most informative chemical properties. The selection of these will be described in the next section, in which we will use a different approach: we will investigate the correlations between specific quantifiable properties of inhibition and specific chemical properties, while we disregard the classification of the compounds. This is necessary if we intend to use all available information, including within-class differences.

**Figure 3:**
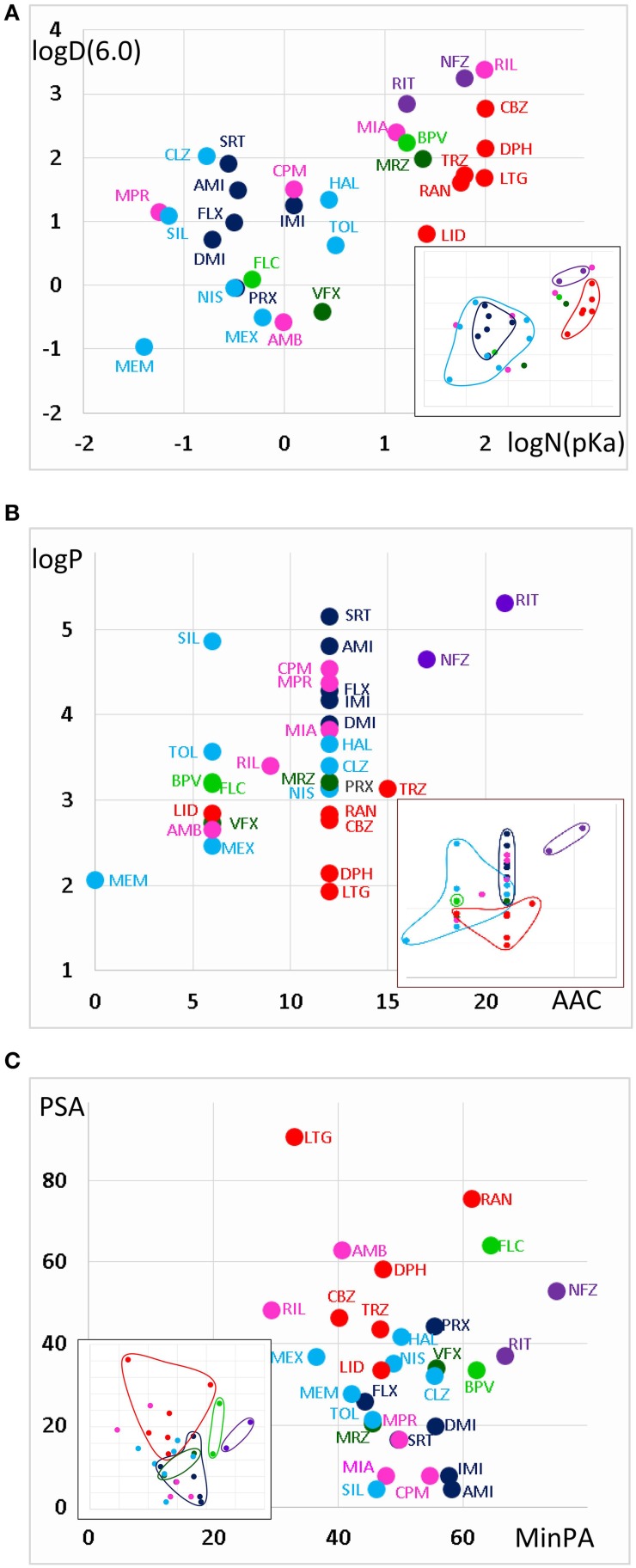
**Position of compound classes within the chemical space**. We attempted to correlate pH-dependent behavior patterns (i.e., Classes A–G) with the chemical properties of the classes (i.e., their location within the chemical space). Classes are color-coded (same colors as in Figure 2), three-letter codes identify individual drugs. The insets are the exact same plots, where we marked the outlines of the areas occupied by specific classes. **(A)** logD(6.3) vs. logN(pKa) plot, **(B)** logP vs. aromatic atom count (AAC) plot, and **(C)** polar surface area (PSA) (**Å**^2^) vs. minimal projection area (**Å**^2^).

The areas inhabited by the classes in three projections of the n-dimensional chemical space are illustrated in Figure 3. In Figure 3A we plotted logD(6.0) values against logN(pKa). Considering the areas occupied by different classes (see inset), we can conclude that predominantly neutral compounds with high logD(6.0) are more likely to manifest Class A or Class E type pH-dependence. Class C and Class F type pH-dependence occurred with predominantly charged compounds, with lower logD(6.0) values. Class B, Class D, and Class G mechanisms were not determined by logN(pKa) or logD(6.0, because compounds belonging to these classes were scattered widely. We need, therefore, to try to find descriptors which can differentiate Class A from Class E, as well as Class C from Class F; and which are able to define Classes B, D, and G.

Figure 3B illustrates logP values plotted against aromatic atom count (AAC) values. Both chemical properties are extremely important in determining the type of inhibition in general (Lenkey et al., 2010, 2011) and of pH-dependence in particular. We can observe that Classes A and E are completely differentiated: Class A contains compounds with relatively low logP (<3.2), and with one or two aromatic rings. Three aromatic rings, and high logP (>4.5), on the other hand, seems to predestine compounds to manifest Class E characteristics (i.e., alkalic pH hinders recovery and decelerates binding/unbinding kinetics). Indeed, aromatic atom count has been found to be a major determinant of reversibility (Lenkey et al., 2010), and of unbinding rate (Smith et al., 2006). The difference between Classes C and F also seems to be established: Class C compounds (i.e., the ones with very low reversibility at alkalic pH) tended to be more lipophilic compounds with two aromatic rings, while Class F compounds were either less lipophilic, or had only one (none for memantine) aromatic rings. A small overlap nevertheless existed between the two groups, which is not surprising, since as we mentioned, haloperidol was occasionally clustered into Class A. More unexpected was the fact that three Class G compounds covered the exact same area as Class C compounds. The two Class B compounds both have one aromatic ring, and they are very close in terms of logP as well, but of course two compounds are too few to reliably define an area in the chemical space. The descriptors logP and AAC still could not help much to localize Class D and Class G compounds within the chemical space.

In Figure 3C, polar surface area was plotted against minimal projection area (the “slimness” of the molecule). This plot gives a hint regarding the requirements for Class B properties: both compounds had minimal projection areas between 60 and 70 Å^2^. Class E compounds were the bulkiest, and Class C compounds were still relatively wide. High polar surface area seemed to predetermine compounds to have Class A or B properties, while low polar surface area favored Class C, F, and G mechanisms. We still have not understood what makes a compound act with Class D properties (i.e., having very low reversibility at acidic pH); unexpectedly it was apparently not strictly determined by pKa, since these values differed considerably for the two compounds (mirtazapine – 7.8, venlafaxine – 8.9). Note, however, that this range is exactly between the range of Class A and Class C compounds. They both had relatively low logP values (3.2 and 2.7), and moderate polar surface areas (20.6 and 33.9 Å^2^), these properties, however do not differentiate them from other classes. Class G is obviously a heterogeneous group of compounds, which have been clustered together because they exhibited no obvious distinguishing features in their pH-dependent inhibition pattern. Heterogeneity of the group, and the low number of compounds prevented proper characterization in terms of chemical properties. Apart from ambroxol and riluzole, which are different from the rest of the class in all important chemical properties, the three Class G compounds which were close to Class C properties in terms of AAC and logP (Figure 3B), and also shared their range along minimal projection area and PSA axes.

We can finally summarize what can be predicted regarding pH-dependent inhibition properties, based on the chemical properties of compounds.

When a large fraction of the molecules is neutral at pH = 7.3 (i.e., pKa < 7.8), they are not very lipophilic (logP < 3.2), and considerably polar (PSA > 33), there is good chance that we will find Class A inhibition, i.e., fast and reversible inhibition which is not much affected by pH.

When moderately low pKa (7.0–8.0) was accompanied with high lipophilicity (logD > 4.6) and aromaticity (more than two aromatic rings), a different pattern emerged. Class E compounds, ritanserin and nefazodone were the only ones where the potency actually decreased at alkalic pH. Onset rates were also decelerated. We reason that while for most compounds alkalization helps to overcome the energy barrier needed for deprotonation, for these highly lipophilic and aromatic compounds the equilibrium is probably shifted toward the membrane-bound neutral form already at pH = 7.3. Further alkalization makes molecules even more reluctant to leave the membrane phase, and therefore at alkalic pH fewer molecules actually enter the internal cavity within the channel, and access the binding site.

Having moderate pKa values also allowed two special inhibition patterns. When it was combined with low lipophilicity, Class D pattern emerged; which essentially means low acidic reversibility. We speculate, that acidification could effectively keep molecules charged within the inner cavity of the channel, thereby preventing their re-enrty to the membrane phase. It is logical that this effect cannot work with molecules that are highly lipophilic, or with molecules that are predominantly neutral even at pH = 6.0. The two compounds that exhibited Class D inhibition had logP values in the same range as Class A compounds, but higher pKa values (not as high though as that of Class C compounds). Nevertheless, from our data Class D properties cannot be unambiguously predicted. Note that some Class A compounds, as well as tolperisone, bupivacaine and ambroxol, also showed compromised recovery at acidic pH, therefore it seemed advisable to investigate correlations without considering which class individual drugs belong to (see next section).

Moderate pKa values also allowed Class B properties (i.e., when alkalization increased potency and also accelerated onset kinetics), when compounds had higher lipophilicity than Class A compounds (logP > 3), were rather bulky (minimum projection area around 60–70 Å2), and contained only one aromatic ring. Class B type of inhibition, nevertheless, was not a “moderate pKa-specific” phenomenon, since the pKa value of flecainide was 9.61. We would suppose that having a single aromatic ring and a high minimum projection area (it was only exceeded by the two Class E compounds; Figure 3C), are more important determinants of Class B pattern. Moderate pKa value with high lipophilicity (bupivacaine), and high pKa value with moderate lipophilicity (flecainide) both seem to allow Class B properties.

When high pKa (> 9) is combined with high lipophilicity (logP > 4), typical Class C properties are expected to occur (low alkalic reversibility, and strong pH-dependence of both apparent affinity and reversibility). There were two atypical Class C compounds as well, desipramine and paroxetine, which showed acceleration of onset kinetics upon alkalization, similarly to Class B compounds. These two compounds had the lowest logP and logD values within their class (Table 1, Figures 3A,B), in which they were similar to flecainide. Their minimum projection area and polar surface area values were also close to those of Class B compounds (Figure 3C). However, when we consider their reversibility at alkalic pH, their difference from Class B is unquestionable, and their affiliation with Class C seems justified. We suppose that the chemical basis of this must be the presence of two aromatic rings.

High pKa and high lipophilicity did not guarantee Class C properties, but also allowed Class F and Class G inhibition patterns. Even if we consider compounds with two aromatic rings, which have pKa > 9 and logP > 3.1, we still have four additional compounds besides the six Class C compounds included in this group: nisoxetine and clozapine from Class F, as well as maprotiline and chlorpromazine from Class G. Nisoxetine and clozapine show inhibition patterns that are indeed very similar to Class C compounds (only their reversibility at alkalic pH is higher), while maprotiline and chlorpromazine had a somewhat different pattern: they had very low reversibility at all three pH values. This indicates that the mentioned chemical properties may allow different patterns of pH-dependence, depending on some additional, not yet identified chemical properties.

The difference between Class C (practically irreversible at alkalic pH) and Class F (moderately reversible) compounds seemed to be that lower logP value or only one aromatic ring seemed to predispose compounds to Class F inhibition pattern.

Finally Class G seemed to be a collection of chemically diverse molecules, which happened to have no obviously distinguishable characteristics in this specific experimental protocol. Although nefazodone was in most trials clustered into this group, we felt it reasonable to discuss it as a member of Class E, because it showed qualitative features identical to ritanserin. Mianserin also had some resemblance to Class E both chemically [see its logD(6.0) and logN(pKa)], and in inhibition properties (slowing of onset rate with increased pH). Riluzole and ambroxol also have been shown to differ in their modes of action from the rest of Class G compounds (Lenkey et al., 2010). The rest of Class G; maprotiline, and chlorpromazine are somewhat similar to Class F compounds both chemically and in inhibition properties, but their reversibility is less pH-dependent. We expect that here again more can be learned from investigation of correlations in an approach that is blind to classification.

### The chemical basis of different aspects of pH-dependent inhibition

**Figure 4:**
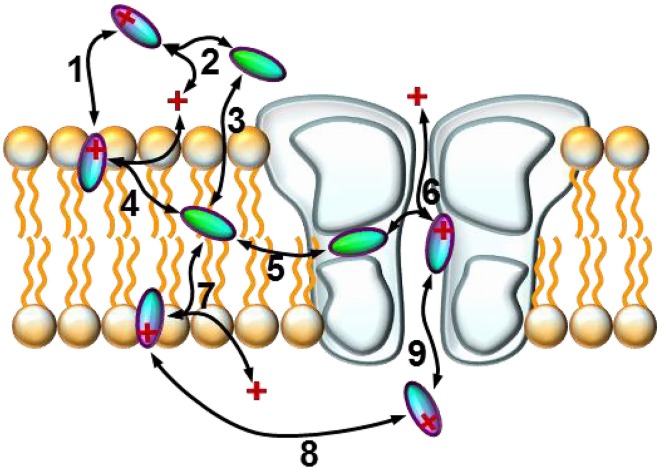
**Schematic model of the sub-processes of drug access**. An illustration of the hypothetical sub-processes of drug access that must be supposed based on experimental data: **(1)** partitioning of the charged molecule at the outer membrane interface; **(2)** protonation/deprotonation in the extracellular space; **(3)** partitioning of the neutral molecule at the outer membrane interface; **(4)** protonation/deprotonation at the outer membrane interface; **(5)** entry/exit through the fenestration (hydrophobic pathway); **(6)** protonation/deprotonation within the inner vestibule; **(7)** protonation/deprotonation at the inner membrane interface; **(8)** intracellular diffusion; **(9)** entry/exit through the activation gate (hydrophilic pathway).

**Figure 5:**
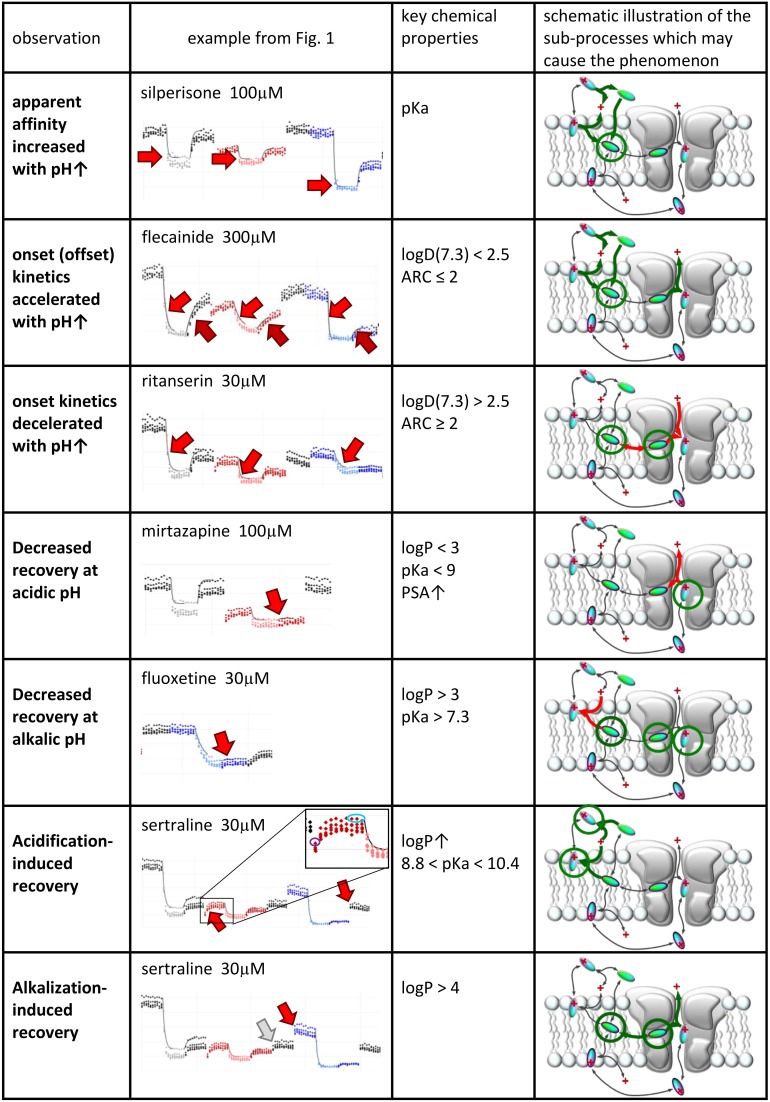
**Suggested contribution of the sub-processes of drug access to seven experimentally detected phenomena**. Seven phenomena of pH-dependence detected in experiments. 2nd column: Illustration of the phenomena on peak amplitude vs. time plots. 3rd column: Chemical properties that are likely to determine occurrence of the phenomenon. 4th column: Sub-processes affected during the occurrence of the phenomena. Green and red arrows indicate accelerated and decelerated sub-processes, respectively. Circled molecules indicate accumulation of drug molecules in that specific position.

We will discuss six major phenomena that could be quantified from the amplitude plots. We attempted to synthesize the observations, and place them into a framework of a general hypothesis regarding the major steps along the access pathway of drugs toward the binding site. All assumed sub-processes of drug access and egress, which may be of different importance depending on the chemical nature of individual compounds are summarized in Figure 4. Our major findings are summarized in Figure 5, which: (in the 2nd column) shows a characteristic occurrence of each observed phenomenon chosen from Figure 1 (or Supplemental Figure 1); lists the major chemical properties which seem to determine their occurrence (3rd column); and shows a graphic representation of their hypothetical explanation (4th column).

#### pH-dependent apparent affinity

**Figure 6:**
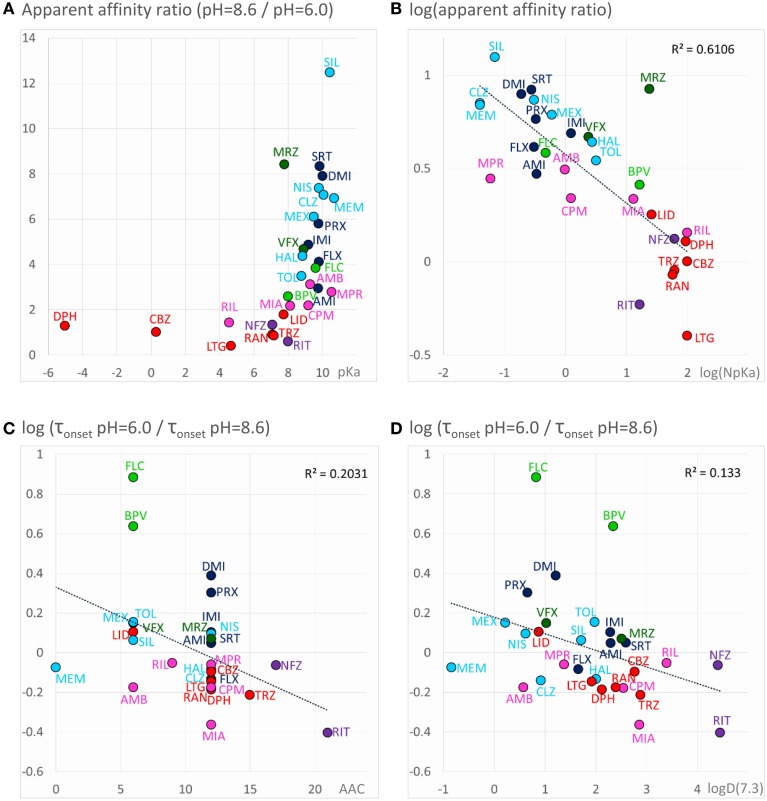
**Chemical properties affecting pH-dependent affinity and onset kinetics**. Correlations of the extent of pH-dependence with chemical properties. Classes are color-coded (same colors as in Figure 2), three-letter codes identify individual drugs. **(A)** Ratios of apparent affinities measured at alkalic vs. neutral solution, plotted against calculated pKa values of the drugs. **(B)** The same correlation could be made close to linear by mathematical transformations: The logarithm of the alkalic/neutral apparent affinity ratio, plotted against the logarithm of the percentage of neutral form at pH = 7.3 [logN(pKa)] (neutral fraction was calculated from pKa values using the Henderson–Hasselbalch equation—see Methods). **(C)** pH-dependent acceleration/deceleration of onset as a function of aromatic atom count (AAC). **(D)** pH-dependent acceleration/deceleration of onset as a function of logD(7.3).

For all predominantly positively charged molecules the affinity was increased at alkalic pH, as it was expected. When the apparent affinity ratios [IC_50_ (pH = 6.0)/IC_50_ (pH = 8.6)] were plotted against pKa values, an abrupt increase was seen at about pKa = 8 (Figure 6A). Seven compounds out of the 30 had pKa values below 7.7, and none of them was significantly more potent at pH = 8.6 (the maximal difference was 1.43-fold). On the other hand, all compounds with pKa value above 8.2 (18 out of the 30) had apparent affinity ratios above 2 (range: 2.17–12.48). The affinity ratio - pKa correlation was evidently not linear, therefore, we also plotted the affinity ratios against the logarithm of the percentage of neutral form at pH = 7.3 (logN(pKa); calculated using the Henderson-Hasselbalch equation as described in Methods). This plot gave a significant correlation (*R*^2^ = 0.49; *p* < 0.001). The correlation coefficient was even higher (*R*^2^ = 0.61; *p* < 0.001) when the logarithm of the apparent affinity ratios was plotted against logN(pKa) (Figure 6B). These results suggest that the apparent affinity is manifestly determined by the ability of drug molecules to populate an intramembranous pool, which is only accessible for their neutral form (Figure 5, 1st row).

#### Accelerated/decelerated onset kinetics

Onset and offset kinetics seemed to change in parallel, but because the offset rate was often occluded by incomplete recovery, it seemed best to concentrate on onset time constants for quantitative evaluation. It was remarkable that the expected significant acceleration of both onset and offset was only observed in the case of two drugs: flecainide and bupivacaine at *p* < 0.01 level. Three additional compounds (mexiletine, paroxetine, and desipramine) showed acceleration of onset at *p* < 0.05 level, but no significant change of offset. For six rapidly acting drugs (carbamazepine, lidocaine, riluzole, ambroxol, silperisone, tolperisone) the acceleration of onset might have gone undetected, because our experimental protocol might not have resolved it. For the rest of the compounds, however, the onset/offset rates were resolvable, but still no acceleration was observed. Three of the compounds, ritanserin (*p* < 0.001), trazodone (*p* < 0.01), and mianserin (*p* < 0.05), even exhibited decelerated onset at alkalic pH. This suggests that the lipophilic (membrane) environment may not only present an energy barrier to overcome, but also an energetically favorable trap along the access route. Searching for chemical properties that could predict the pH-dependence of onset rates, we found the highest correlation with aromaticity (aromatic atom count or aromatic ring count), and with logD values (at pH = 7.3 or 8.6) (Figures 6C,D). There is a significant correlation between aromaticity and logD [e.g., *R*^2^ = 0.52; *p* < 0.001 between aromatic atom count and logD(7.3)], therefore one cannot be sure which of these two correlations represents a genuine causal relationship. It can be observed, nevertheless, that acceleration of the onset of inhibition due to alkalic pH was only seen for compounds with two or less aromatic rings, and logD(7.3) lower than 2.5, while for compounds with two or more aromatic rings, and logD(7.3) higher than 2.6 the onset tended to slow down. In the case of the former group, the dominant effect must have been the accelerated crossing of the lipophilic barrier due to deprotonation (Figure 5, 2nd row), while for the latter (more aromatic, more lipophilic) group, the deceleration was probably due to being trapped in a lipophilic environment (Figure 5, 3rd row). For the compounds where the effect of pH was evident on their apparent affinity, but not on the onset and offset kinetics, we suppose that these two opposing effects may have counterbalanced each other.

#### Decreased recovery at acidic pH

**Figure 7:**
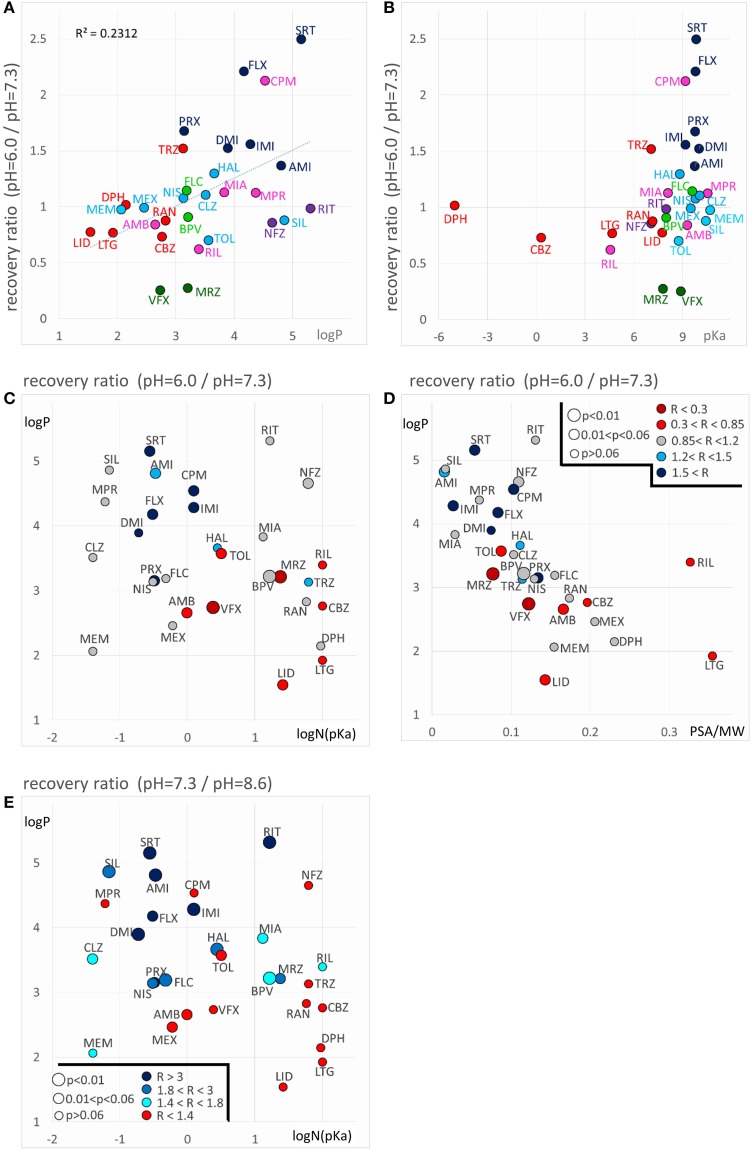
**Chemical properties affecting pH-dependent reversibility**. Correlations of acidification- and alkalization-induced changes in recovery with chemical properties. **(A)** Ratios of acidic (pH = 6.0) vs. neutral (pH = 7.3) recovery are plotted against logP and **(B)** against pKa. In both panels classes are color-coded (as in Figure 2), three-letter codes identify individual drugs. **(C)** Acidic/neutral recovery ratios are color coded, and plotted on the logP against logN(pKa) plane, and **(D)** on the logP against polar surface area/molecular weight (PSA/MW) plane. Light and dark red indicates moderately and strongly decreased recovery, respectively; gray indicates no change; light and dark blue indicates moderately and strongly increased recovery, respectively. [See **(D)** for color codes. “R” stands for “ratio”). The size of the data points indicates the level of significance, as it is also shown in **(D)**. **(E)** Neutral/alkalic recovery ratios are color coded and plotted on the logP against logN(pKa) plane. Light to dark blue indicates increasing ratios, red indicates minimal or no change. Levels of significance are coded by the size of data points. Codes are shown in the lower left corner.

Decreased recovery at acidic pH was not at all general even among the predominantly positively charged molecules. Only two compounds: venlafaxine and mirtazapine showed significantly (*p* < 0.01) decreased recovery at pH = 6.0, while the differences for nefazodone (*p* = 0.024), ambroxol (*p* = 0.036), lidocaine (*p* = 0.046), tolperisone (*p* = 0.057), and carbamazepine (*p* = 0.075) were non-significant at *p* < 0.01 level. The tendency was more frequent with drugs which are relatively hydrophilic (logP < ~3), and which have a substantial neutral fraction at pH = 7.3 (pKa < 9; N(pKa) > 1%; Figures 7A,B). We can also observe that (the predominantly charged) Class C compounds showed an opposite effect; acidic pH helped recovery from the inhibition by these drugs. In order to see the combined effects of lipophilicity and charged-neutral ratio, we plotted logP values against logN(pKa) (Figure 7C). Color code shows the ratio of acidic/neutral recovery values: light and dark red indicates moderately and strongly decreased recovery, respectively; gray indicates no change; light and dark blue indicates moderately and strongly increased recovery, respectively. The size of the data points indicates the level of significance. It seems that the compounds that are prone to be trapped at pH = 6.0 are more hydrophilic, and/or have a substantial neutral fraction at pH = 7.3 which becomes protonated at acidic pH (Figure 5, 4th row). Some of the “decreased-acidic-recovery” compounds, such as riluzole, lamotrigine, and carbamazepine, however, cannot be protonated even at pH = 6.0. All three compounds have, however, an exceptionally high number of hydrogen bond acceptors and donors, as well as polar surface compared to their size (Figure 7D). This suggests that the effect of external pH may go beyond changing the charged/neutral ratio of ligands, and may also affect protein-ligand interaction by other ways, possibly via interfering with hydrogen bonds or van der Waals forces. This chemical environment, which seems to be able to trap hydrophilic, polar or charged molecules must be different from the lipophilic trap mentioned above, we suggest that it is located within the inner vestibule of the channel.

Until now we have discussed the compounds that had a lower recovery at acidic pH than at neutral pH. A somewhat different, but equally reasonable question would be to ask which compounds had low recovery at acidic pH, irrespectively of their reversibility at neutral pH. From Figure 2B and Table 2 we can see that some compounds have low reversibility at all three pH values. There are five compounds with reversibility below 0.6 at all three pH values: sertraline, ritanserin, nefazodone, maprotiline, and chlorpromazine. The most obvious property of this group is that they all have very high logP values. In terms of pKa they are rather heterogeneous, although they all have a substantial charged fraction at pH = 7.3, and are predominantly charged at pH = 6.0. We will return to discussing this group in section “Alkalization-induced Recovery.”

#### Decreased recovery at alkalic pH

Decreased recovery at alkalic pH, was much more common, especially for more lipophilic drugs with relatively high pKa (Figure 7E). Essentially all compounds which are predominantly charged at neutral pH (pKa > 7.3), and are highly lipophilic (logP > 3) exhibited decreased recovery at pH = 8.6 at an at least *p* < 0.05 significance level. The difference was 1.49- to 5.07-fold as compared to pH = 7.3; see the three shades of blue in Figure 7E. (In the case of the only exception, tolperisone, the difference was 1.35-fold, with *p* = 0.05.) The reason why maprotiline and chlorpromazine were exceptions is, that these compounds already had very low recovery even at pH = 7.3 (Supplemental Figure 1). For predominantly neutral compounds it is expected that alkalic pH would not change their reversibility (red points: no significant change, or less than 1.4-fold change). Interestingly, it seems that it was not enough to neutralize a substantial fraction of the molecules by alkalic pH, in order to show low reversibility they also needed to be lipophilic. This must have been the reason while memantine, mexiletine, ambroxol, and venlafaxine failed to exhibit strongly decreased recovery at pH = 8.6. This indicates that the environment where molecules are trapped at alkalic pH must be apolar and lipophilic. We reason that high lipophilicity and high pKa (i.e., being predominantly positively charged) counteract each other, the former helps the accumulation of compounds in this lipophilic environment, from which access to the binding site is easy, while the latter retains molecules in the outer, charged layer of the membrane, from where the binding site is not accessible. In such situation this sensitive balance can be easily shifted by alkalization of the extracellular fluid, when more molecules are allowed into the lipophilic environment; or by acidification, when more molecules are retained (Figure 5, 5th row).

#### Acidification-induced recovery

Observing the plots of peak amplitudes (Supplemental Figure 1), we can note that the incidents of external pH change may also be of interest. Although in these points no inhibitor was given or washed out, when channels had not recovered fully from prior drug effects, we often saw unmistakable signs of enhanced recovery produced by pH change alone. We had four such points of pH change in our solution exchange protocol: neutral-to-acidic, acidic-to-neutral, neutral-to-alkalic, and alkalic-to-neutral transitions. We observed enhanced recovery for specific subsets of the compounds at all four transitions. Interestingly, some of the drugs reacted with enhanced recovery to both acidification and alkalization.

Acidification-induced recovery: In the absence of inhibitor, exchange from neutral (pH = 7.3) to acidic (pH = 6.0) solution caused an instantaneous drop of the amplitude to 72.4 ± 1.6% of the control value. The effect of acidic pH on sodium currents is a well-known phenomenon, thought to be due to channel block by protons (Woodhull, 1973). Upon acidic-to-neutral exchange the inhibition instantaneously and fully recovered. No significant change in the amplitude was observed at neutral to alkalic or alkalic to neutral transitions.

**Figure 8:**
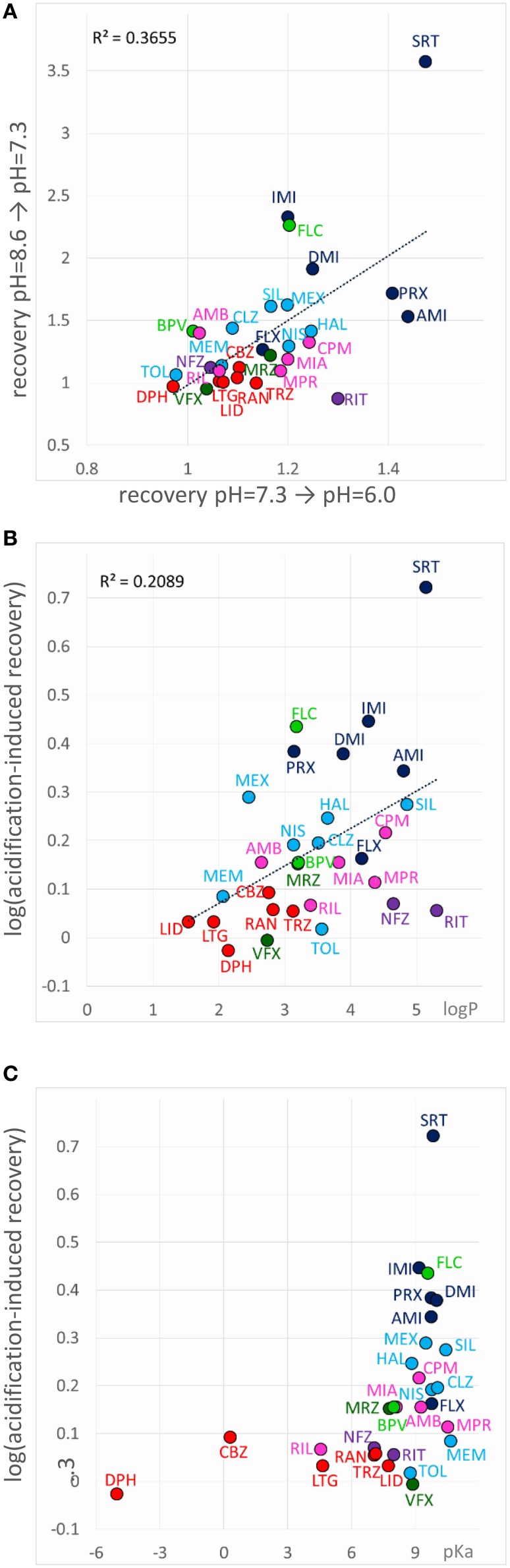
**Chemical properties affecting acidification-induced recovery**. **(A)** Correlation between recovery caused by alkalic-to-neutral and neutral-to-acidic solution exchange. **(B)** Acidification-induced recovery plotted against logP and **(C)** pKa. The sum of the logarithms of the two recovery steps (neutral-to-acidic and alkalic-to-neutral) was used as a measure of acidification-induced recovery.

When an inhibitor had previously been perfused at neutral pH, and the recovery was not complete (mostly Class C, F, and G compounds), neutral-to-acidic solution exchange evoked a component of recovery. We quantified this by calculating the ratio of current amplitudes evoked at the end of this section vs. the amplitude of the first current evoked in acidic solution (circled in the inset shown in the 6th row of Figure 5). The explanation is essentially the same as in the case of “decreased recovery at alkalic pH” (see above), except that in this case the recovery must be compromised even at neutral pH. Lowering the pH then helps the protonation of the compounds, which shifts the “protonated-in-the-outer-membrane-layer-pool” vs. “deprotonated-within-the-membrane-pool” equilibrium, and also increases aqueous solubility, helping partitioning into the aqueous phase, and therefore wash-out is accelerated. Most, but not all compounds, which showed enhanced recovery upon neutral-to-acidic exchange reacted similarly to alkalic-to-neutral exchange (both are shown by red arrows in the 6th row of Figure 5); there was a significant correlation (*p* < 0.001) between the reactions of compounds to these two pH decreasing steps (Figure 8A; Supplemental Figure 1). Decreasing the pH must have helped escape from the lipophilic trap, therefore this phenomenon would be most evident for predominantly charged, lipophilic compounds. Indeed, logP had a significant correlation (*p* < 0.01) with the extent of acidification-induced recovery (Figure 8B), and there was a definite optimum of pKa for it between 8.8 and 10.4 (Figure 8C). In these Figures 8B,C acidification-induced recovery was calculated by the sum of the logarithms of the two recovery steps. We can conclude that as we have expected, essentially the same chemical properties predispose compounds both to “decreased recovery at alkalic pH” and to “acidification induced recovery.”

#### Alkalization-induced recovery

Alkalization-induced-recovery could in principle be monitored at incidents of acidic-to-neutral (gray arrow in 7th row of Figure 5), and neutral-to-alkalic (red arrow in 7th row of Figure 5) transitions. Calculating from the former, however, is impracticable, because acidic-to-neutral transition reflects the combination of three effects. For example for all Group A compounds but sertraline the amplitude becomes larger upon re-application of neutral medium after acidic medium perfusion. This may be due to alkalization-induced recovery occurring upon acidic-to-neutral transition, but also to acidification-induced recovery, which have occurred at the beginning of perfusion by acidic medium. In addition, removal of proton block also occurs concurrently. For this reason, it is difficult to judge the true extent of increase due to alkalization-induced recovery alone. There is no such complication at the neutral-to-alkalic transition, which, we believe, truly reflects the effect of alkalization.

**Figure 9:**
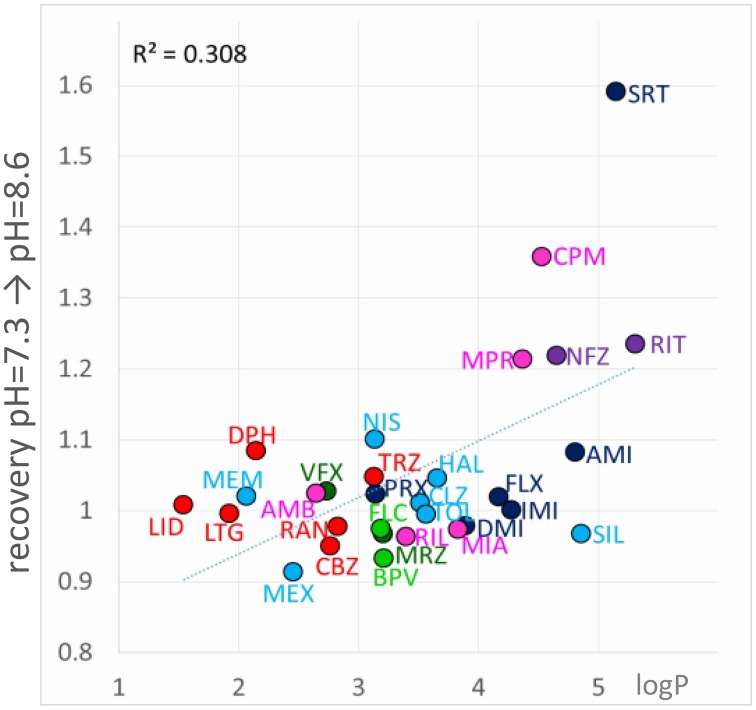
**Chemical properties affecting alkalization-induced recovery**. Neutral-to-alkalic solution exchange-induced recovery plotted against logP.

If “acidification-induced-recovery” was essentially equivalent with “decreased alkalic recovery,” then one could expect that “alkalization-induced-recovery” should be equivalent with “decreased acidic recovery,” and therefore we should best see alkalization-induced-recovery with compounds that are prone to accumulate in the hydrophilic trap, i.e., relatively hydrophilic, polar drugs with a relatively low pKa. This, however, was absolutely not what we found. In fact the compounds that showed “decreased acidic recovery” (either a tendency or a significant difference) failed to display at the same time “alkalization-induced recovery” (with the only exception of nefazodone). These typically small, polar, relatively hydrophilic compounds, with a substantial neutral fraction at pH = 7.3, were fully released by neutralization, and alkalization did not further help their escape. The compounds that needed alkalization to be released, were to be found in the “low acidic recovery” group, these compounds had low reversibility at both acidic and neutral pH, and therefore, perfusion of neutral medium could not release them fully. As we have mentioned, they were chemically different from the “decreased acidic recovery” group, most of them being highly lipophilic (Figure 9). Of the compounds with the 7 highest logP values, 5 showed prominent alkalization-induced recovery (sertraline, nefazodone, ritanserin, maprotiline, and chlorpromazine). These are the exact same compounds, which had low reversibility values (<0.6) under all three pH conditions. In the case of nisoxetine (logP = 3.14) the phenomenon was small and only significant at *p* = 0.05 level. We suppose that for this group of compounds both lipophilic and hydrophilic interactions contributed to binding. The finding that high lipophilicity was a requirement for both acidification and alkalization-induced recovery suggests that the same lipophilic drug may exhibit both phenomena. Indeed, all six compounds showed enhanced recovery both upon neutral-to-alkalic and alkalic-to-neutral transitions (Supplemental Figure 1).

The fact that both alkalization and acidification can help the recovery of some compounds suggests that accumulation of drug molecules can occur simultaneously in at least two distinct pools, which represent different chemical environments. It is possible that for full recovery, drug molecules need to take two consecutive steps, one requiring deprotonation (this might be the step of entering the membrane phase from the binding site; i.e., steps #6 and #5 in Figure 4) and the other protonation (this might be regaining the charge, and/or partitioning to the aqueous phase; i.e., steps #2, #4 and #1 in Figure 4). The rate-limiting step may be either, or both. It is also possible that alternative binding sites co-exist for protonated and for neutral forms of compounds. We assume that the high incidence of non-monotonic pH-dependence of the recovery (Figure 2B), and the occasional occurrence of non-monotonicity even in the apparent affinity plots (Figure 2A) may also be due to this “double accumulation” phenomenon.

## Discussion

Of the three major propositions from the literature of pH-dependent action of sodium channel inhibitors, we have confirmed and refined one, and re-specified two:

First, our data confirmed that the extent of pH-dependence of potency is dependent on the ratio of charged/neutral forms of the molecules, and that in general alkalization increases potency of partially charged compounds. However, we have noticed that extremely high lipophilicity and aromaticity may override this tendency: For such compounds we hypothesize that alkalization may prevent their entry into the aqueous environment of the inner cavity, thereby decreasing their potency. In addition, the relatively weak pH-dependence of lidocaine was somewhat surprising; this points to the importance of the pKa value of compounds. We propose that the calculated pKa value of drugs (such as local anesthetics) may be used to predict how much its effect will be resistant to acidification of the tissue, and that it is advisable to use compounds with low enough pKa in situations where tissue acidification may be a problem.

Next, we observed that the onset kinetics was not always accelerated by alkalization. The major predictors of the pH-dependence of kinetics were pKa, lipophilicity and aromaticity. Onset kinetics of predominantly positively charged compounds (with a high pKa) would be accelerated by alkalization, because deprotonation helps these molecules enter the membrane phase. However, highly aromatic and lipophilic compounds may find the membrane phase an energetically favorable environment, which may slow down their access to the binding site. The two effects may counterbalance each other, acceleration is dominant with less lipophilic compounds, and deceleration is dominant for lipophilic compounds (especially if they also highly aromatic) with relatively low pKa, i.e., which do not depend on alkalization for effective partitioning into the membrane phase.

Finally, although acidification hindered recovery for some of the drugs, it was much more common that recovery was hindered by alkalization. The former phenomenon was common among relatively hydrophilic and polar compounds with low pKa value, while the latter was a characteristic of highly lipophilic, predominantly charged compounds.

The complex effect of pH on onset/offset kinetics and recovery suggest the existence of at least two chemically different pools where drug molecules can be accumulated. Drugs (especially the ones that have a substantial neutral fraction at pH = 7.3) can be trapped by protonation, probably in a polar environment (which could be the aqueous cavity within the channel itself), and can be rescued by alkalization. Drugs that are strongly lipophilic, and are predominantly charged at neutral pH can be trapped after deprotonation in an apolar environment (which may be the membrane, or an apolar sub-region at the channel-membrane interface), and can be rescued by acidification. Finally, some of the most lipophilic drugs show low reversibility at both acidic and alkalic pH, and both alkalization and acidification may help the rescue of a fraction of the molecules. The supposed major steps of drug access and egress are illustrated in Figure 4. When the external pH is changed, many of the sub-processes can be affected, depending on the chemical nature of the drug molecule. The scheme we propose can account for all diverse phenomena observed in the experiments, as it is specified in the schematic figures of the right column of Figure 5.

The conclusion of our study shows the importance of the membrane phase in the apparent affinity and the onset/offset kinetics of sodium channel inhibitors. It is a common mistake to attribute slow onset and recovery of inhibition to the slow inactivated state preference of the inhibitor (Karoly et al., 2010), when it is equally possible that the rate of onset and offset is limited by drug-membrane interactions. The complexity of ligand-membrane interactions as shown by this study suggests that these interactions may be as important as ligand-protein interactions in determining the mode of action of sodium channel inhibitors, and ultimately their therapeutic applicability. Therapeutic profiles of individual drugs are in general determined by their target spectrum, and within a target class, their subtype selectivity. This is somewhat different in the case of sodium channel isoforms, where the key residues involved in drug binding are conserved across all mammalian isoforms, therefore, their binding region is practically identical (Mike and Lukacs, 2010; Nardi et al., 2012). Indeed, most of the efforts during the last two decades to develop truly isoform-selective compounds (i.e., with more than 10-fold selectivity) have failed so far, only Nav1.8 selective compounds have been successfully developed (Clare, 2006; Lenkey et al., 2011; Nardi et al., 2012). For other isoforms, the minimal inter-subtype selectivity is overshadowed by the huge state-selectivity of typical sodium channel inhibitors: affinities between different conformational states may differ by several orders of magnitude (Lenkey et al., 2010, 2011). Therefore, the essence of the therapeutic applicability of sodium channel inhibitors is thought to be “functional selectivity,” not subtype selectivity. Functional selectivity is the direct consequence of the mode of action, therefore investigation of specific modes of action is a key to successful drug development strategies (Nardi et al., 2012).

The complexity and heterogeneity of drug-membrane interactions points to two interesting aspects of sodium channel inhibitor action. One is the question of alternative binding sites, the other is the role of membrane composition in the potency of sodium channel inhibitors.

It is quite possible that compounds with so widely different chemical properties may have different binding sites on the channel. The fact that extracellular pH had a profound effect even on highly lipophilic compounds, indicates that for these compounds the most favorable position is probably at the membrane interface. Accumulation of compounds at this position has already been shown in molecular dynamics simulations for isoflurane, benzocaine, and phenytoin (Raju et al., 2013; Martin et al., 2014), which are polar but not charged compounds. Having charged compounds accumulated in this position may modify channel function indirectly by modifying membrane structure and electric charge density, and directly by interacting with the voltage-sensor. Similar mechanisms have been shown for lipid modulation of ion channels (e.g., Hite et al., 2014; Zaydman and Cui, 2014). It is possible, therefore, that inhibitor molecules may modulate sodium channel gating even without entering the inner vestibule. Earlier we have proposed the presence of alternative binding sites (Mike and Lukacs, 2010), based on the wide chemical variety of inhibitor molecules, and on the exceptional pharmacological promiscuity of sodium channels (Huang et al., 2006; Lounkine et al., 2012; Zhang et al., 2015). Molecular dynamics studies indeed confirmed the presence of multiple energetically favorable positions for sodium channel inhibitor compounds (Raju et al., 2013; Barber et al., 2014; Boiteux et al., 2014; Martin and Corry, 2014) within the channel. It is also possible, however, that compounds which are highly lipophilic and at the same time have a high pKa (i.e., not easily deprotonated), and therefore probably accumulate at the membrane interface near the channel, may modulate channel gating via an interaction with the voltage sensor domains.

The second aspect is, that if this position is indeed energetically favorable, than the composition of the membrane is a major determinant of the potency of these drugs. There is plenty of evidence for the importance of ligand-membrane interactions in determining drug potency for transmembrane proteins (see Box1 in Morris et al., 2012). It is therefore conceivable that specific sodium channel inhibitor drugs show preference for pathological membrane composition, as it has been proposed (Morris et al., 2012), or even show selectivity among cell types or subcellular compartments depending on their membrane composition. These ideas suggest that a deeper understanding of ligand-membrane interactions may lead to development of therapeutically more specific inhibitor molecules.

### Conflict of interest statement

The authors declare that the research was conducted in the absence of any commercial or financial relationships that could be construed as a potential conflict of interest.

## Supplementary material

Supplemental Figure 1 **Peak amplitude vs. time plots for all 30 compounds**. Amplitude plots show averaged normalized amplitudes of five individual experiments. Thin black lines show average of the five exponentials fit to individual curves. 2D chemical structure of drug molecules is also shown.

Supplemental Table 1 **Measured properties of inhibition for all 30 compounds at 3 pH values**. The first 15 columns are the same as in Table 2 of the main text. In addition, the calculated ratios, and the significance levels are also given.
