# Supplementary material for: Different pH-sensitivity patterns of 30 sodium channel inhibitors suggest chemically different pools along the access pathway
Source: Front Pharmacol. 2015 Sep 25;6:210. doi: 10.3389/fphar.2015.00210 (PMC4585259; doi:10.3389/fphar.2015.00210)

Supplemental Fig. 1

Class A

CBZ 300

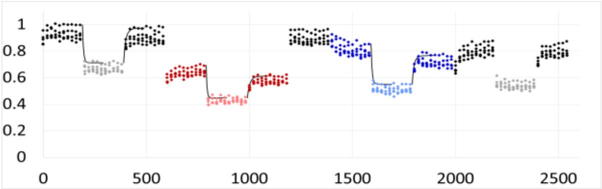

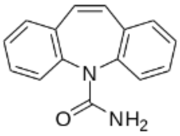

LTG 300

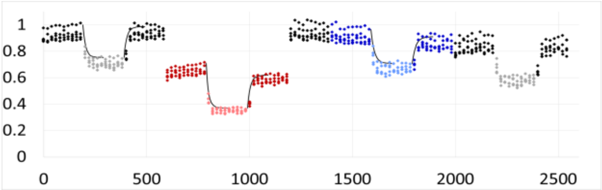

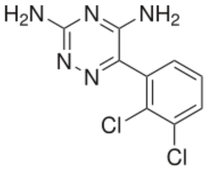

DPH 300

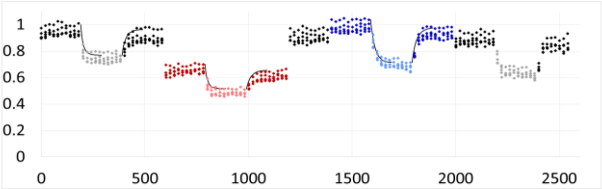

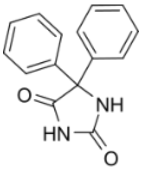

TRZ 100

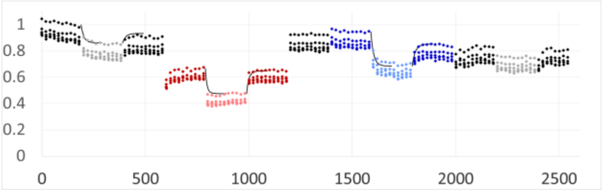

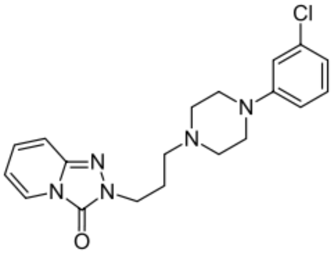

RAN 100

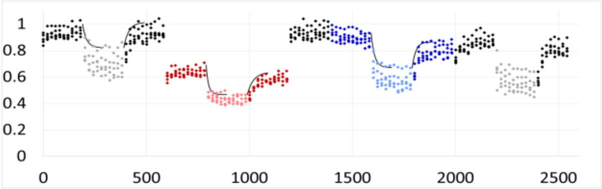

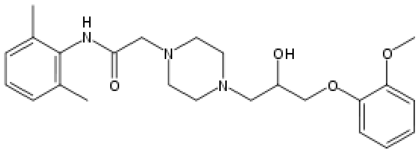

LID 300

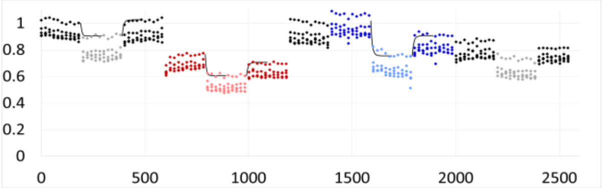

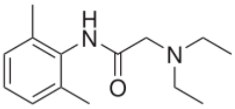

LID 1000

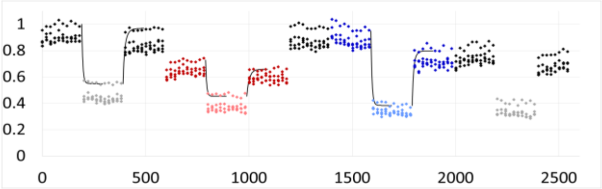

Class B

BPV 100

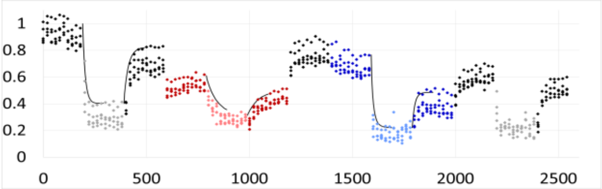

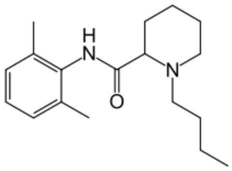

FLC 300

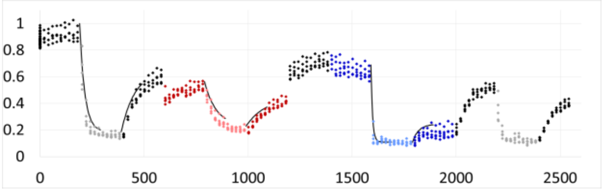

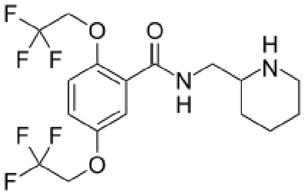

Class D

MRZ 100

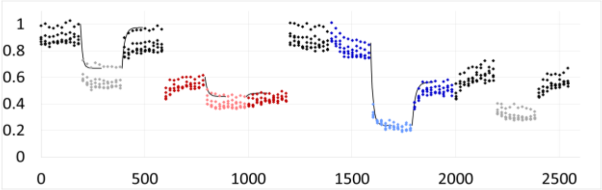

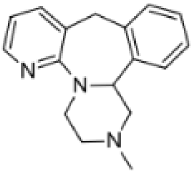

VFX 100

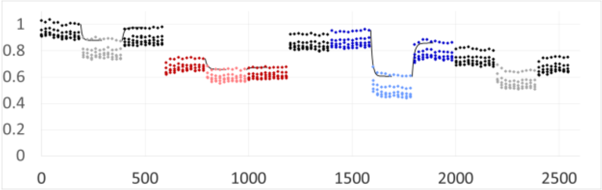

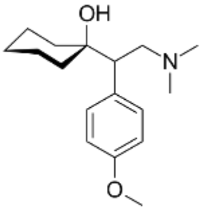

Class E

RIT 30

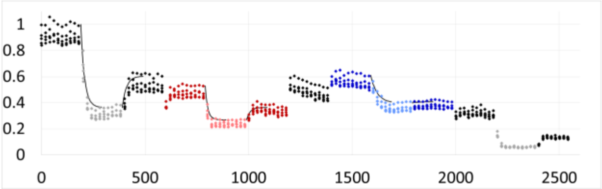

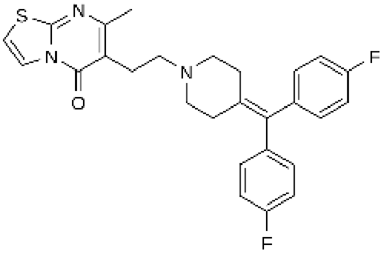

Class C

FLX 30

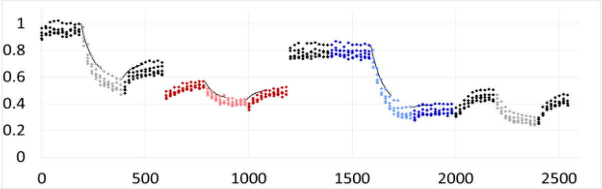

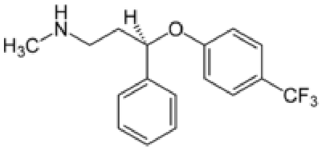

SRT 30

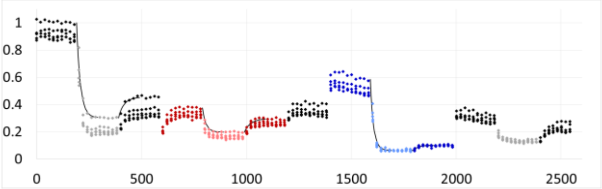

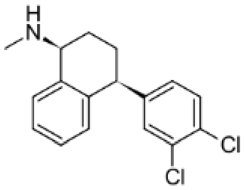

PRX 30

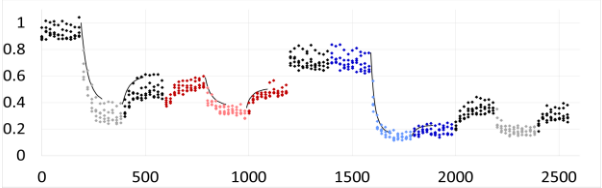

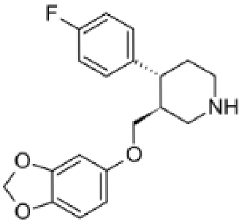

AMI 30

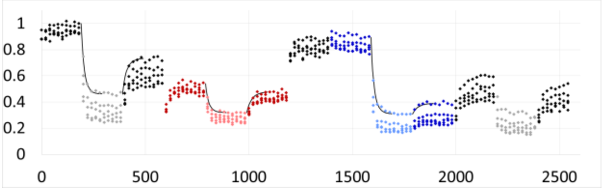

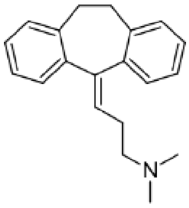

IMI 30

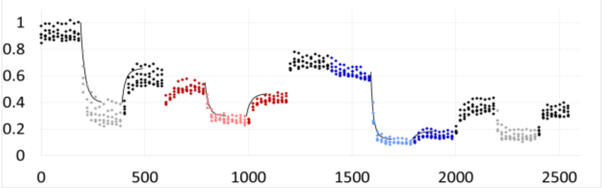

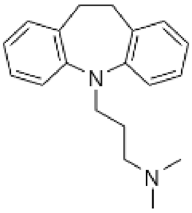

DMI 30

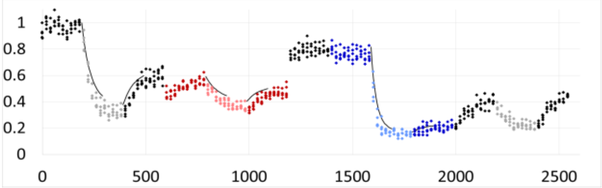

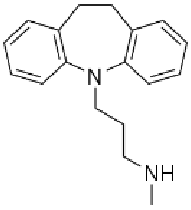

Class F

HAL 30

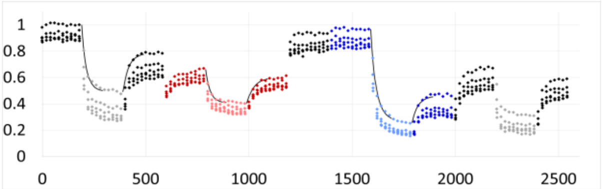

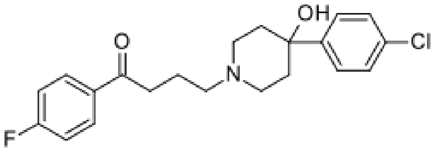

NIS 100

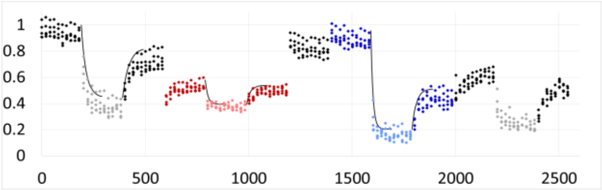

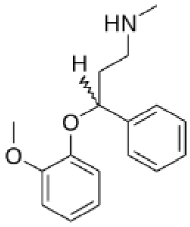

CLZ 100

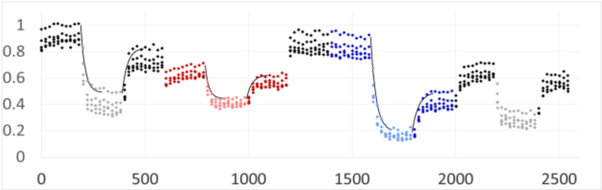

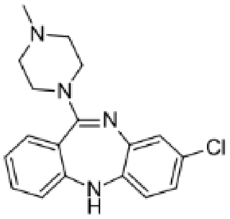

MEM 30

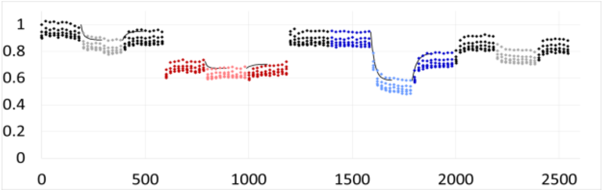

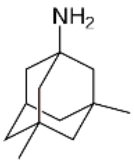

MEM 100

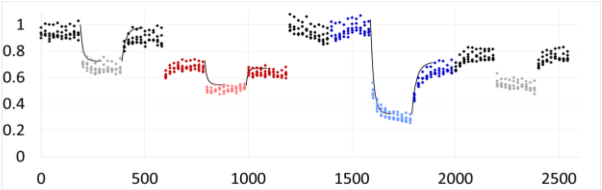

MEX 300

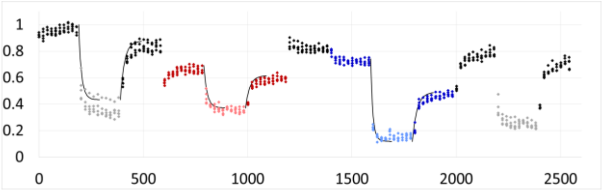

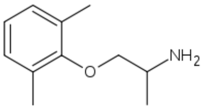

SIL 100

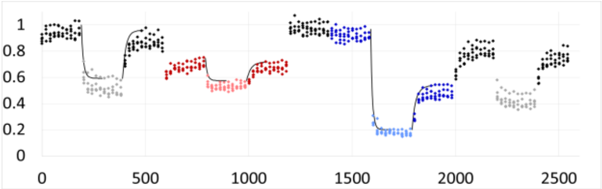

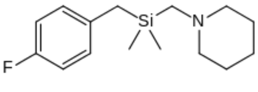

TOL 100

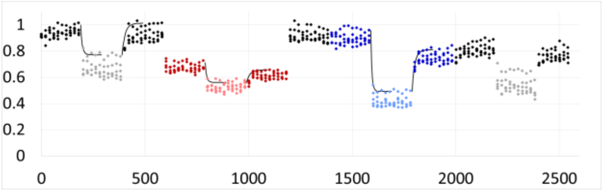

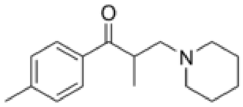

Class G

MPR 10

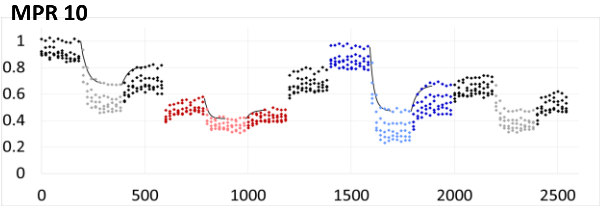

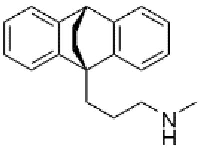

MIA 30

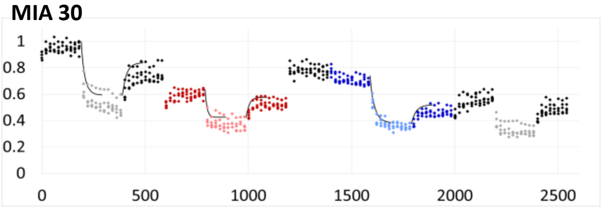

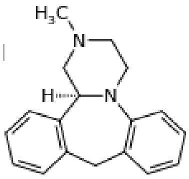

CPM 30

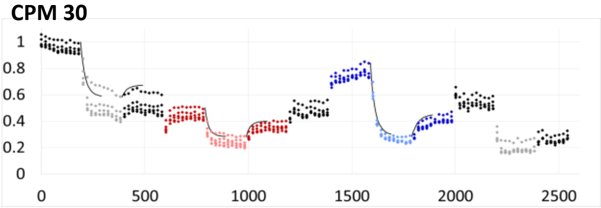

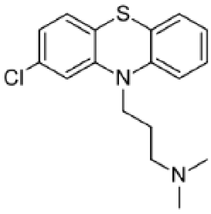

RIL 30

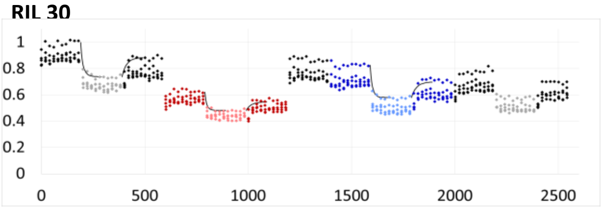

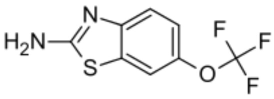

AMB 100

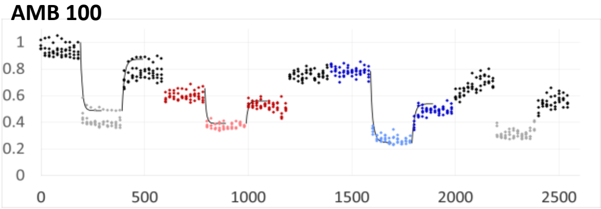

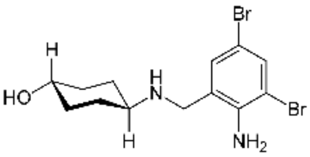

NFZ 30

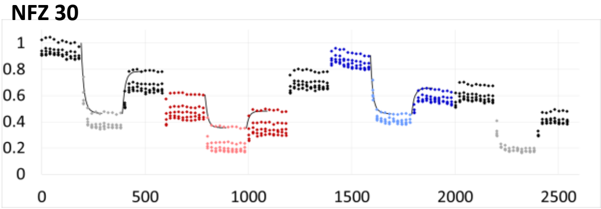

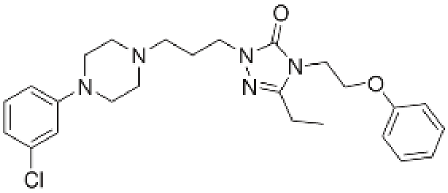

Supplement: Supplemental Figure 1 — Peak amplitude vs. time plots for all 30 compounds. Amplitude plots show averaged normalized amplitudes of five individual experiments. Thin black lines show average of the five exponentials fit to individual curves. 2D chemical structure of drug molecules is also shown. [file Image1.PDF]
